# Circularly Permuted Fluorescent Protein-Based Indicators: History, Principles, and Classification

**DOI:** 10.3390/ijms20174200

**Published:** 2019-08-27

**Authors:** Alexander I. Kostyuk, Aleksandra D. Demidovich, Daria A. Kotova, Vsevolod V. Belousov, Dmitry S. Bilan

**Affiliations:** 1 Shemyakin-Ovchinnikov Institute of Bioorganic Chemistry, Moscow 117997, Russia; 2 Pirogov Russian National Research Medical University, Moscow 117997, Russia; 3 Institute for Cardiovascular Physiology, Georg August University Göttingen, D-37073 Göttingen, Germany

**Keywords:** genetically encoded biosensors, circularly permuted fluorescent proteins

## Abstract

Genetically encoded biosensors based on fluorescent proteins (FPs) are a reliable tool for studying the various biological processes in living systems. The circular permutation of single FPs led to the development of an extensive class of biosensors that allow the monitoring of many intracellular events. In circularly permuted FPs (cpFPs), the original N- and C-termini are fused using a peptide linker, while new termini are formed near the chromophore. Such a structure imparts greater mobility to the FP than that of the native variant, allowing greater lability of the spectral characteristics. One of the common principles of creating genetically encoded biosensors is based on the integration of a cpFP into a flexible region of a sensory domain or between two interacting domains, which are selected according to certain characteristics. Conformational rearrangements of the sensory domain associated with ligand interaction or changes in the cellular parameter are transferred to the cpFP, changing the chromophore environment. In this review, we highlight the basic principles of such sensors, the history of their creation, and a complete classification of the available biosensors.

## 1. Introduction

At present, autofluorescent proteins (aFPs) have become indispensable tools in many biological and medical studies. The first protein from this group called green fluorescent protein (GFP) was isolated from the jellyfish *Aequorea victoria* in 1962 [1] and subsequently characterized [2,3,4,5,6,7,8]. To date, a wide color variety of fluorescent proteins has been developed from different organisms [9,10,11], including representatives of other species such as Anthozoa [12], copepods [13], and even chordates [14].

The main reason for the wide applicability of aFPs is their ability to auto-catalytically form chromophores without requiring any additional factors [15]. Therefore, they can be expressed in different cellular systems maintaining their optical properties. Many modern analytical techniques exploit the unique nature of these macromolecules in order to directly visualize structures and processes in living cells and organisms [10]. Owing to their increasing scientific demand, different types of aFPs with optimized parameters—such as fluorescence intensity, maturation rate, phototoxicity, and oligomeric state—have been engineered using molecular biology methods [10].

An extremely promising direction in modern research is the development of genetically encoded fluorescent indicators (GEFIs) based on aFPs that can be used to visualize and quantify enzymatic activity and conformational state of proteins as well as changes in the concentrations of particular molecules or biophysical parameters in vivo, including living cells, tissues, or whole organisms [16]. In general, GEFIs can be described as chimeric constructions based on at least one FP, the optical properties of which depend on a cellular parameter of interest. Therefore, GEFIs convert biochemical events into macroscopic visible signals that can be detected using standard optical equipment. Unlike traditional analytical approaches, GEFIs provide the following advantages. Being protein molecules, they are non-invasive and can be targeted to different cell types or even subcellular compartments, and, unlike chemical dyes, they are not prone to leaking from the cells during imaging. Thus, GEFIs have become the primary choice in most experiments requiring real-time registration of biochemical parameters in living systems.

The variety of GEFIs developed to date reflects the importance of this technology and difficulties arising in attempts to develop a full classification serve as the best confirmation of this fact. The most general classification of these indicators based on architecture reveals several groups: (1) single FP-based sensors without individual sensory domains; (2) single FP-based sensors with individual sensory domains; (3) Forster resonance energy transfer (FRET)-based sensors; (4) dimerization-dependent (dd)FPs-based sensors; (5) proximity imaging (PRIM)-based sensors; and (6) bimolecular fluorescence complementation-based sensors. The choice of the specific design depends on the precise objective of a study; hence, probes for measuring pH [17], halide anions [18], Ca^2+^ [19], hydrostatic pressure [20], and redox environment [21] may be developed on the basis of the direct interaction between an analyte and the chromophore or modified surface of a FP. Despite the many advantages of these probes, such as relatively simple design, low molecular weight, and ample targeting opportunity, the range of cellular events that can be investigated using this approach is limited.

Development of GEFIs for other cellular parameters usually requires considerably more genetic engineering efforts, often implying the creation of chimeric proteins with additional domains resulting from natural ‘sensors’. In such probes, the sensory unit is responsible for the detection of the tested parameter and provides a molecular switch that affects the FP structure, and thus the optical properties. Novel FRET- [22,23,24], ddFPs- [25,26,27,28], and PRIM-based [29,30,31] probes have been used to constantly expand our knowledge regarding metabolic fluxes and signaling pathways that occur in living cells; however, they have limitations that arise from the fact that aFPs have quite rigid structure protecting the chromophore from external environmental shifts. Non-ideal coupling between sensory and reporter units often leads to small maximal response amplitudes, especially if the former does not show high conformational rearrangements during functioning. Herein, we describe how circularly permuted (cp)FP-based sensors help to overcome these obstacles, highlight the general principles underlying their development, and provide a classification of these probes by analytes measured and color.

## 2. Circularly Permuted aFPs as Reporter Units for GEFIs

Since the discovery of GFP, researchers have been studying its biophysical properties for its application in investigations of both fundamental and applied science. Crystallographic studies revealed that the polypeptide chain of GFP-like proteins forms a rigid monolithic β-barrel consisting of 11 β-sheets that accommodate an internal distorted helix [32,33], which is resistant to conformational changes. The chromophore group maturates autocatalytically on the basis of three conserved amino acid residues located within this region during cyclization and subsequent oxidation [15]. Given that the optical properties of the chromophore strongly depend on its microenvironment, developing GEFIs by creating molecular switches affecting the latter is possible. However, aFPs seemed to have evolved in order to protect the chromophore from shifts in external medium that resulted in its unusual stability to protease-mediated degradation and temperature-induced denaturation [6,34]. Although these features allow the successful expression of functional aFPs in almost all cell types, they significantly hamper the development of GEFIs by preventing efficient conformational coupling with sensory units. Moreover, the natural termini of aFPs that might be fused to sensory units are located in relatively flexible regions far from the chromophore group, which further complicates the situation. Therefore, attempts to develop GEFIs by introducing FPs with native topology into even flexible proteins showing large structural rearrangements often result in sensors with low dynamic range. For example, the voltage indicators FlaSh [35] and SPARC [36] designed on this principle are characterized by fluorescence changes of only approximately 5% and 0.5% per 100 mV, respectively.

Abedi and colleagues first attempted to insert protein sequences into the GFP structure [37]. At that time, phage display and yeast dihybrid system had become important tools for constructing large protein libraries for understanding molecular interactions in biology; however, the first approach could not be applied to in vivo research, whereas the second one was limited to considerably large interfaces. The authors suggested that the rigid structure of GFP could provide a scaffold for upholding an inserted peptide conformation in the manner analogous to the variable domain of an immunoglobulin. The latter consists of a beta-barrel and two loops forming hypervariable regions, and thus shows some similarity to FPs [38]. This was the reason why GFP was chosen as a candidate along with the fact that its fluorescent properties potentially facilitate library properties estimation and following manipulation. For example, checking whether a specific cell type expresses a member of the library or biasing the latter toward sequences with higher expression rate becomes easier by using sorting methods. In their study, 10 candidate positions for insertions were tested, and 3 of them were found to be tolerable to inclusion of variable peptides without pronounced decline in GFP brightness [37].

Subsequently, Doi and Yanagawa reported about a GEFI for TEM1 β-lactamase (Bla) and β-lactamase-inhibitory protein (BLIP) interaction that was developed by inserting the former into position 172–173 of the GFP sequence [39]. This position was discovered by Abedi et al. [37] and selected because of its spatial proximity to the chromophore group, which could provide sufficient coupling. This design was chosen because Bla does not undergo pronounced conformational rearrangements after binding to BLIP [40] and was inspired by the findings of studies showing that the activity of a specific enzyme bearing a foreign peptide was affected by an antibody for that epitope [41,42]. In this particular case, two rounds of random mutagenesis led to the formation a functional GFP::Bla-1 probe for which the fluorescent signal changed in the presence of BLIP, but not in the presence of bovine serum albumin [39]. FRET-based cameleons had already been reported before this study [43]; however, calmodulin is a very flexible protein. Thus, GFP::Bla-1 was one of the first examples to show that even small conformational rearrangements could be converted into detectable optical signals by engineering a molecular switch located close to the chromophore group [39].

The process of circular permutation involves fusing of the natural termini of a protein with a peptide linker, while new termini are being formed in another region of the sequence. In some cases, circular permutation can be achieved through a series of posttranslational modifications such as those performed for bovine pancreatic trypsin inhibitor [44]. However, in most cases, all procedures are performed at the gene level. Initially, circularly permuted proteins were engineered to investigate protein folding. These initial studies supported the concept that, in many cases, the amino acid sequence of a protein and not the natural location of the termini orchestrates corrective spatial organization as functionally active circularly permutated versions of several proteins with native tertiary structures were developed, such as extended phosphoribosyl anthranilate isomerase from yeast [45], aspartate transcarbamoylase [46], the SH3 domain of α-spectrin [47], chymotrypsin inhibitor 2 [48], and thiol/disulfide oxidoreductase DsbA from *Escherichia Coli* [49]; and several other proteins. Although the natural termini of FPs are located in close proximity, which is necessary for circular permutation, it might seem that the monolithic rigid beta-barrel of FPs cannot be successfully subjected to this procedure. Nevertheless, two studies published almost at the same time showed that circularly permuted fluorescent proteins (cpFPs) could retain optical properties [50,51]. As expected, most part of the effective break points were found in the loops connecting the β-sheets; however, some positions in the latter were also tolerant to circular permutation [51]. Subsequent biochemical tests revealed that the obtained permutants are often more pH-dependent, have longer maturation range, and require lower temperatures for folding than their native counterparts. This can be attributed to the fact that the structure of circular permutants is more flexible than that of the native protein, and their chromophores are more accessible to the external media. Another important aspect is that often successful permutation and insertion points overlap.

What does it mean for GEFIs development? One of the studies mentioned above showed that ECFP, EGFP, and EYFP could be subjected to calmodulin insertion at position Y145, which lies in a close proximity to the chromophore [51]. All chimeras were found to be calcium-sensitive, with the EYFP-based variant showing 7-fold enhancement in fluorescence after saturation with Ca^2+^, which exceeded the response level of all other GEFIs for the same analyte available at that time [51]. Subsequently, the described probe (similar to the GFP::Bla-1 sensor discussed above [39]) was named Camgaroo; its topology was used to develop other sensors, for example, Camgaroo-2 for calcium [52]; Green cGull for cGMP [53]; Flamindo [54], Flamindo2 [55], and Pink Flamindo [56] for cAMP; GINKO-1 for potassium [57]; and green and red non-permutated intensity-based glutamate-sensing fluorescent reporters (iGluSnFRs) for glutamate [58]. Although this review does not discuss these indicators in detail, we need to specify that all of them work on the principle that is extremely analogous to that of cpFP-based probes wherein effective conformational coupling is achieved by FP structure destabilization and locating a molecular switch close to the chromophore with a mobile microenvironment. Another field of cpFPs implementation, that we do not describe precisely, arises from the fact that maintaining the main optical properties circularly permuted versions demonstrate different relative positions of the chromophore and the termini [51]. Therefore, they allow the optimization of the FPs interaction efficiency in FRET or PRIM indicators as it becomes possible to “rotate” fluorescent proteins without affecting the general structure of the probe [29,59,60,61,62,63,64].

One of the important requirements for the development of an indicator bearing Camgaroo-like topology is the proximity of the sensory unit termini, which is an often, but not ultimate feature, of natural proteins. Therefore, using a cpFP as a flexible reporter module is considerably convenient. The initial cpFP-based GEFIs—G-CaMP [65] and pericams [66]—were independently engineered on the basis of a fragment of myosin light chain kinase M13 and calmodulin. Their outstanding features served as the best proof-of-principle and, in the next two decades, this approach was implemented for the development of a wide range of GEFIs for various molecules and cellular events that have been discussed below.

Next, we intend to highlight the main advantages and disadvantages of cpFP-based probes over their counterparts, mainly FRET-based probes. Their most important benefit is the high dynamic range attributed to the efficient conformational coupling between sensory and reporter units, as described above. Moreover, even proteins with relatively modest conformational rearrangements can be tested as sensory units, providing an affordable signal-to-noise ratio for measurements [67]. Second, they occupy a narrower part of the light spectrum, facilitating multiparameter imaging when several biochemical events are simultaneously monitored in a single living system. This is extremely important for understanding the interplay between fast processes when combining data from independent experiments becomes difficult [68]. cpFP-based probes have lower molecular weight, which is better for optimizing expression rates and subcellular targeting. They also do not suffer from the difference in pH sensitivities or maturation rates of two FPs by the contrast to FRET-indicators that might mimic specific response.

**Figure 1:**
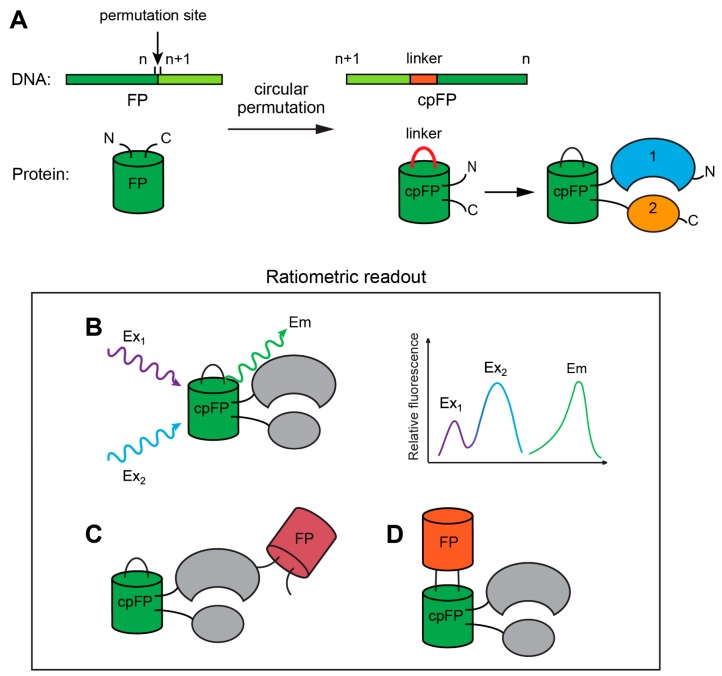
Basic principles of cpFP-based biosensors. (**A**) The scheme of FP circular permutation at the DNA and protein levels. The resulting cpFP can be integrated between the interacting units (1 and 2) of the sensory domain. Units 1 and 2 can be different proteins, subunits or domains of the same protein. (**B**–**D**) Variants of cpFP-based biosensors with ratiometric readout. (**B**) The ratiometric biosensor can be developed on the basis of a single cpFP, since some are characterized by the presence of two peaks in the fluorescence excitation spectrum. (**C**) A second FP with different spectral properties can be attached to the N- or C-termini of a biosensor. (**D**) (Green-Orange)-Matryoshka cassette for designing ratiometric biosensors.

However, biosensors developed using circular permutation have some drawbacks attributed to the same structural features that are responsible for their advantages. First, shifts in spectral properties can be mediated by external factors, including medium acidity and small ion concentrations [51]. This is because of the effective interaction of the chromophore with the environment. Thus, cpFP-based sensors often require the implementation of appropriate pH controls during the course of imaging. Second, the emission effectiveness of cpFPs largely depends on the fusing partners and linker composition, unlike FPs that have a natural topology. Thus, development of these probes is more labor-intensive, and achieving brightness is extremely difficult, unlike for non-permuted versions. Third, such probes usually require more time for maturation, which is faster at low temperatures [51,69]. Finally, many of them are intensiometric, which hampers the accurate quantification of the measured data. Different approaches have been used to overcome this obstacle. For example, a second FP can be fused to the one of the probe termini or inserted into cpFP as an internal control [70,71,72,73]. The basic principles of GEFIs are presented in Figure 1.

We now discuss the main requirements for the reporter part of cpFP-based GEFIs. The ideal candidate for the development of the reporter part of the sensor is a bright permutant, as this parameter of FP is essential for in vivo studies. The usage of many indicators is remarkably limited in tissues owing to the lack of fluorescence intensity. The most vivid are proteins having emission maxima in the green and yellow areas of the spectrum, and proteins that fluoresce in cyanic and red or far-red parts are relatively dim [10]; therefore, development of bright red fluorescent proteins (RFPs) and their permutated versions is desirable. In general, having a wide range of biosensors with different colors is necessary for their implementation in multiparameter imaging [68].

To date, several approaches have been used for constructing libraries of possible protein permutants with consequent screening for the best versions [74,75,76,77]. RFPs represent an area of elevated interest for many researchers, as they are more convenient to use for in vivo studies. Longer excitation wavelengths (500–580 nm) can penetrate deeper into biological tissues, causing less background autofluorescence and less phototoxicity. Moreover, the absorption and autofluorescence of biomolecules such as melanin and hemoglobin in the 450–500 nm region complicates the excitation and detection of signals emitted by biosensors based on yellow and green FPs [78]. Circularly permutated versions of yellow and green FPs were obtained first; hence, historically, most fluorescent biosensors are based on these proteins. The pH dependence of fluorescence, spectral properties, and total fluorescence intensity depend mainly on the reporter unit of the sensor; hence, its replacement by a circular permutant of red fluorescent protein can lead to the elimination of at least some of these shortcomings.

## 3. General Principles for Developing cpFP-Based GEFIs

The development of any genetically encoded cpFP-based biosensor requires the selection of an appropriate sensory unit. The sensory unit is usually a protein or protein domain that undergoes pronounced conformational rearrangement induced by changes in the cellular parameter of interest. In addition, the knowledge that two individual proteins can change binding character under some circumstances can be used to develop probes as it was performed while developing probes such as GCaMPs [65], Pericams [66], cAMPr [79], and ExRais [28]. For selecting a sensory unit, the following factors need to be considered. First, the protein should be able to bind the analyte with high specificity. However, some types of probes can bind to different forms of analyte with comparable affinity. In this case, the biosensor reports concentration ratio, for example, Perceval for [ATP]:[ADP] [80] ratio registration or SoNar for [NADH]:[NAD^+^] ratio [81].

Second, the crystal structure of the apo and ligand-bound forms of the selected protein should be available. The structure can be obtained using either X-ray analysis or NMR spectroscopy. However, if the structure of the sensory unit is not solved, the crystal structures of the homologous proteins can be used as was used for dLights [82], GACh probes [83], iGluSnFR [84], or iGABASnFR [85]. Finally, the structure of the sensory unit should contain regions with detectable conformational mobility. As the latter increases, the likelihood for effective conformational coupling also increases; however, the cpFP-based topology is applicable even to proteins with a very modest flexibility [67]. Once a suitable sensory unit has been determined, the biosensor can be designed.

The commonly used approach to construct a biosensor is a rational design, which involves several steps. First, flexible regions in the structure of the sensory unit are selected. Next, several positions for cpFP insertion are selected in each flexible region. When choosing cpFP insertion positions, it is important not to affect amino acid residues involved in the binding of the analyte. In some cases, inserting deletions or using homologous forms with shorter flexible regions is beneficial as it possibly increases the efficiency of conformational coupling. The first and second approaches were implemented for developing SoNar [81] and ASAP1 [86], respectively. Another method involves the construction of chimeras, as was used for GACh probes, where a long flexible loop of human muscarinic acetylcholine receptor was substituted with a short one from β-2 adrenergic receptor [83]. In any case, checking different homologs, if possible, is effective as some of them might fail to express or respond and others might provide different kinetic or dynamic properties [82,83,85,86,87,88,89].

After appropriate positions have been selected, choosing peptide linkers between sensory and reporter units is necessary. The selection of linkers is a creative task and depends on the proteins selected. For example, a commonly used variant of peptide linkers is available for biosensors based on cpYFP: Ser-Ala-Gly from the N-terminus of cpYFP and Gly-Thr from the C-terminus of cpYFP. These peptide linkers were first used for designing ratiometric probes for Ca^2+^ sensing, such as Pericam [66], and then in probes such as HyPer for H_2_O_2_ [90], RexYFP for NADH/NAD^+^ [91], and CF98 for citrate [92]. For red biosensors, longer linker sequences are usually used (three amino acid residues each from the N- and C-terminus of the permutant). However, a consensus sequence of peptide linkers is not yet available for red sensors.

Occasionally, individual peptide linkers are not used for biosensor design; instead, the N- and C-terminal amino acid residues of cpFP are modified. This approach was used in designing FGBP—biosensor for glucose monitoring in living cells [93]—or FlicR1 for voltage detection [94]. Using special peptide linkers is not always necessary, for example, in sensors such as FHisJ [95] for histidine detection and in the sensor for phosphonates [96] they are absent. However, in both the studies, versions with deletions of several amino acid residues of the sensory unit near the cpFP were tested which represents another strategy for linker region optimization.

After the sensor versions are designed, genetic engineering is performed to express these sensors in bacterial or other systems, and one or several variants with the best fluorescence intensity and response amplitude are selected. Another important characteristic of biosensors is the saturating concentration of analyte. The value of this parameter should be physiologically relevant, i.e., it should be close to the concentration of analytes in the system in which the biosensor is intended to be used. Thus, expected analyte concentration also needs to be considered when selecting the best version of biosensors. In some cases, selecting versions with different kinetic constants or affinity is beneficial as different situations require probes with varying sensitivity [82,87,88,89,97,98,99,100,101,102].

Occasionally, the best biosensor versions selected for several parameters might not deem suitable for use in living systems. For instance, biosensors may have weak fluorescence, low response amplitude, or analyte concentrations to which the sensor responds do not lie in the physiological range. In this case, the sensor needs to be further optimized.

Random mutagenesis is often used for sensor optimization. Random mutations can be introduced along the entire length of a chimeric protein, or only in the sensory or fluorescent parts. Peptide linkers can also be optimized by randomizing their amino acid sequences, as was used for red H_2_O_2_ indicator HyPerRed [103]. In this approach, the linkers length may also vary. Linkers optimization is often required for color change as different reporter modules rely on different coupling mechanisms [102,103]. In general, random mutagenesis is a complicated and time-consuming procedure, as numerous sensor variants need to be analyzed.

In addition to random mutagenesis, structure-guided protein engineering strategy is used. For example, this method was applied for the optimization of the [ATP]:[ADP] ratio reporter Perceval [80] (the optimized version was called Perceval HR [104]). In that study, mutagenesis was performed around the ATP-binding pocket in order to change the affinity of the indicator to adenylate nucleotides. The rational mutagenesis was also used for the development of HyPer3 [105], an improved variant of HyPer. Some other examples of probes designed using rational mutagenesis are mentioned in the following sections.

Most of the existing biosensors have been designed using a rational approach. However, a new library-based method—domain-insertion profiling with sequencing (DIP-seq)—has recently been proposed [106]. In this method, libraries of sensor structures are created by randomly inserting the reporter unit into the sequence of the sensory unit by using transposons. Because only forward, in-frame, and correctly folded cpFP insertions result in fluorescent constructs, libraries are then enriched using fluorescence-activated cell sorting in the presence of analytes, and then next-generation sequencing is performed. Once an enriched library is obtained, constructs from the sorted libraries are expressed and tested for in vivo biosensor activity.

**Figure 2:**
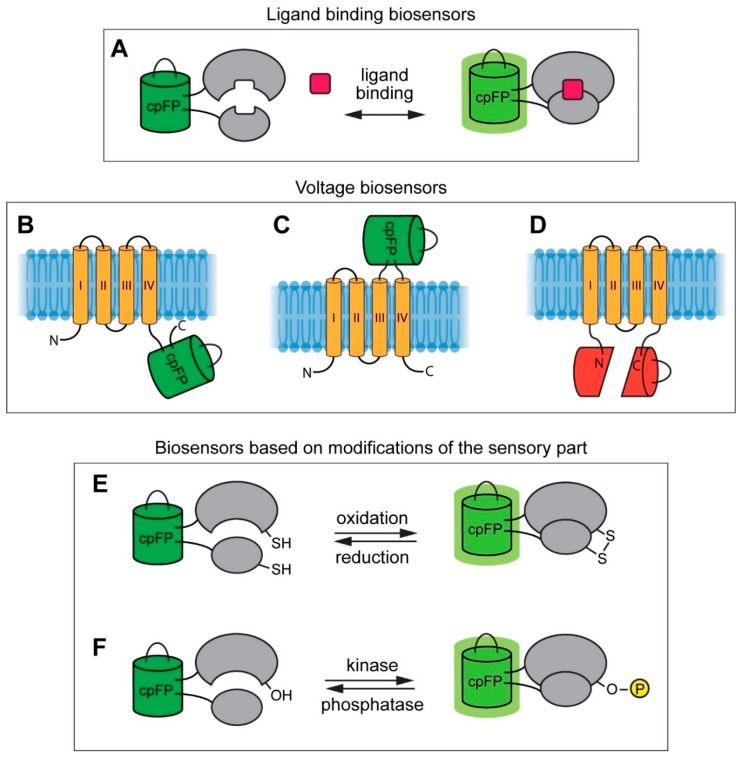
Main types of cpFP-based biosensors. (**A**) cpFP-based biosensors with sensory domains capable of ligand binding. (**B**–**D**) Voltage biosensors. The principle is based on the fact that membrane depolarization causes a movement in transmembrane voltage sensitive domain (VSD), which ultimately leads to conformational changes in a cpFP, changing its fluorescence intensity. cpFP may be (**B**) fused to the terminus of a VSD or (**C**) integrated into the region between transmembrane segments of a VSD. (**D**) N- and C-termini of VSD may be fused with a cpFP whose polypeptide chain is split into two parts. These separate parts form a mature protein. (**E**,**F**) cpFP-based biosensors with sensory domains capable of undergoing protein modifications: (**E**) oxidation, (**F**) phosphorylation.

By using this method, the authors showed that active insertion sites were not exclusively found in the areas of remarkable conformational changes, which are usually considered for cpFP insertion during the rational design of biosensors. Thus, DIP-seq allows the identification of numerous variants for active biosensors, unlike in the rational approach [106]. Different types of GEFIs are presented in Figure 2.

## 4. Classification of cpFP-Based GEFIs by Analyte Measured and Color

### 4.1. GEFIs for Measuring Inorganic Ions

Ca^2+^ is one of the most important secondary messengers in cell signaling pathways. It plays key roles in numerous cellular processes such as fertilization, development, learning, and memory [107]. At present, many excellent small-molecule calcium indicators with suitable properties in terms of kinetics and fractional fluorescence change are available [108]. However, the process of introducing these molecules into tissues is invasive and nonspecific. Furthermore, loading of molecules is destructive and incompatible with long-term imaging. The use of FP-based indicators can overcome these problems. Various genetically encoded calcium indicators (GECIs) with a wide range of colors; different K_d_, appropriate for each cell compartment; and pKa, are available (Table 1).

The first cpFP-based GECI (G-CaMP: the chimeric protein consists of M13-peptide, CaM, and cp148GFP) was developed by Nakai and co-workers [65]. Almost at the same time, Pericams were constructed from cp145EGFP, M13-peptide, and CaM [66]. The subsequent version of the Ca^2+^ sensor had a larger dynamic range (8.5-fold vs. 4-fold). Moreover, Nagai et al. showed that the introduction of subtle mutations in the amino acids close to the chromophore remarkably changed the Ca^2+^-dependent behavior of biosensor. The substitution of phenylalanine for tyrosine at residue 203 (Y203F) led to the creation of a ratiometric sensor—ratiometric-Pericam [66]. The mechanism underlying this type of sensors is the interaction between the M13 and CaM mediated by Ca^2+^ binding. This conformational shift modulates the environment of the cpGFP chromophore by allowing the formation of new domain interfaces, rearrangement of protein side chains, and reduction of solvent access to the chromophore, leading to an increased fluorescence owing to water-mediated cooperation between the chromophore and R377 of CaM [109,110]. At present, the GCaMP family of intensiometric Ca^2+^ sensors is one of the most widely used tools for monitoring Ca^2+^ concentration fluctuations, especially in neurons [111].

More than any other cell types, neurons are critically dependent on spatially and temporally controlled Ca^2+^ dynamics. This is achieved via an exquisite level of compartmentalization of Ca^2+^ storage and release from various organelles. Thus, further evolution of calcium sensors developed in the direction of increasing sensitivity to neuronal activity [112,113,114]. GECIs have been used to measure the dynamics of large populations of neurons over weeks, even during learning [112,114,115]. They can track activity in local and long-range axonal projections and provide information about the activity of neurons that are inaccessible to electrophysiological methods [116].

Large-scale mutagenesis and screening approaches allowed the generation of excellent Ca^2+^ sensors called G-CaMP6 and G-CaMP8 with greater dynamic range (11.4 and 38, respectively) and sensitivity (K_d_ = 158 and 200 nM, respectively) than those of the respective small-molecule-based probes [113]. Moreover, GECIs tagged with target peptide sequences allowed the imaging of Ca^2+^ dynamics in specific organelles [117]. For instance, the spatiotemporal properties of calcium-measuring organelle-entrapped protein indicators (CEPIA) with greater K_d_ (368–558 µM) than that of G-CaMP6s (144 nM) allowed the resolution of Ca^2+^ import into individual mitochondria while simultaneously measuring ER and cytosolic Ca^2+^ levels [118].

Expanding the color options for Ca^2+^ sensors led to the creation of blue- and red-emitting variants [119]. Since GFP and RFP chromophores are significantly different, the development of red calcium indicators has progressed slower than that of the green analogs. Nevertheless, R-GECO, based on cp-mApple [119], R-CaMP2 [120], and photoconvertible calcium sensor based on cp-mMaple, named GR-GECO [121], have been successfully applied for in vivo imaging owing to their lower phototoxicity and autofluorescent background and deeper tissue penetration, allowing the labeling of specific types of cells such as neurons and astrocytes and targeting to subcellular compartments. Importantly, the mechanism of the red calcium sensor R-GECO1 is slightly different from that of GCaMP, which has been described above. In R-GECO1 Lys78 from strand eight of cpmApple, adjacent to the circular permutation site, forms an ionic interaction with the phenolate oxygen of the chromophore and is stabilized by a hydrogen bond from Ser62, immediately following the M13pep-cpmApple linker [122]. Conformational rearrangements in the sensory unit affect these interactions providing a molecular switch.

Despite all these advantages of calcium sensors, they have one main drawback—all calcium indicators act as calcium buffers. Therefore, the expression of any type of GECI may unintentionally change the spatiotemporal dynamics of this essential secondary messenger [123]. Recently, several attempts have been made to reduce the buffering activity of FRET-based GECIs. One of them involves the use of troponin C protein from swim bladder and white muscle of *Opsanus tau* [124]. The general idea was to develop a sensory unit with one or two calcium binding sites per indicator molecule. To that end, a minimal calcium binding motif was engineered on the basis of EF-hand from C-terminal domain of troponin C. The resulted probes (Twitch sensors) bind less calcium ions compared to their counterparts that can lead to reduced ion buffering during long-term expression. Moreover, because troponin C is muscle-specific, GECIs such as Twitch sensors would not be affected by interactions with endogenous proteins when expressed in non-muscle tissues.

Obtaining the crystal structures of Ca^2+^-bound bright states for some variants such as GCaMP2 [109,110], GCaMP5 [114], and GCaMP6 [125] might provide a structural basis for Ca^2+^-induced fluorescence changes and promote the rational design of improved GECI. Detailed information is listed in Table 1.

At present, the repertoire of genetically encoded fluorescent sensors has been expanding for not only monitoring common metabolites and secondary messengers, but also a rather unusual class of molecules present in our body—transition metals (TMs). TMs play essential roles in numerous biological processes, including respiration, gene transcription, enzymatic catalysis, and cell signaling; however, their excessive or inappropriate accumulation may become toxic [133].

Most genetically-encoded TM sensors for zinc and copper, as well as heavy metals, including lead and cadmium, were developed using the metal-binding motif in combination with one or two FPs (Table 2). The evolution of TM sensors shows that the first genetically encoded sensors were FRET sensors. The Cu(I) sensor was created by introducing cysteine-rich metallothionein-like Cu(I)-sensing domains of Atm1 between a FRET pair [134]. Subsequently, Choi et al. developed a new genetically encoded fluorescent Cu(II) sensor GCS-2 by the rational insertion of the Cu-binding tripeptide ATCUN (the amino terminal and Cu- and Ni-binding motif) into cpGFP [135]. This sensor has been used to detect dynamic Cu(II) fluctuations on the surfaces of live mammalian cells, representing, to our knowledge, the first report for Cu(II) imaging by using a genetically encoded fluorescent sensor.

The situation is the same for Zn(II) sensors. When several versions of Zn(II) FRET sensors had been constructed (ZapCY1 [136] and ZapOC2 [137]), Palmer et al. constructed a single FP-based, genetically encoded Zn(II) sensor GZnP1(Kd = 580 nM) that consisted of two zinc fingers from *Saccharomyces cerevisiae* Zap1 fused to a cpGFP [138]. ZnGreen2 (K_d_ of 20 mM for Zn(II)) was constructed by connecting a zinc hook peptide from Rad50 to each of the two termini of cp-monomeric teal FP [139]. It has lower affinity for zinc, allowing the monitoring of intracellular Zn^2+^ dynamics.

The rapid increase in biosensors of this type has revealed many interesting features of TMs. These biosensors help to monitor the actual concentrations of Cu and Zn(II) in different cell compartments, to detect heavy metals in organisms and to study their interaction with other proteins. However, genetically encoded TM sensors still have several limitations in brightness, dynamic range, and kinetics, unlike GECIs, and need to be improved.

Furthermore, a unique class of biosensors was engineered using the so-called “Matryoshka technology”—it is a generalized platform to create dual-FP biosensors with large dynamic ranges by using a single FP cassette, named GO (green–orange) Matryoshka [73]. The cassette nests a stable reference FP (large Stokes shift mOrange) within a reporter FP (cp-GFP). GO-Matryoshka yields green and orange fluorescence under blue excitation. This technology allowed the conversion of existing single-emission biosensors into ratiometric versions, namely, calcium sensors (MatryoshCaMP6s) and ammonium transport activity sensors (AmTryoshka1;3). The Matryoshka approach provides an alternative design for ratiometric sensors with a large dynamic range and is particularly advantageous when terminal fusion of FPs is not tolerated. Detailed information is listed in Table 2.

### 4.2. Genetically Encoded Voltage Indicators

Living cells precisely regulate their membrane potentials as they orchestrate biochemical events crucial for normal metabolism. Ion fluxes, enzyme activity, as well as protein/protein and protein/lipid interactions strongly depend on the difference between intercellular and extracellular potentials, which not only is important for maintaining the microenvironment that is tuned for appropriate cellular machines functioning, but also provides an opportunity to set regulation switches for adjusting intracellular biochemical processes in response to environmental changes [140]. This principle is implemented to a greater extent in the nervous system where neurons transmit signals to each other by affecting membrane potentials of target cells, eventually generating complex animal behavior [141]. Detailed investigation of this issue has been performed using traditional approaches such as patch-clamping and voltage chemical dyes; however, despite their contribution, to our knowledge, these methods are invasive and often do not provide the desired spatial resolution. Therefore, developing instruments that would enable the registration of voltage shifts in compartments, such as small-caliber dendrites, spines, and boutons for individual cells in the real-time mode, are required. Such studies can be conducted by implementing GECIs, but calcium fluxes might not necessarily reflect membrane potential changes because of different temporal parameters and kinetic constants of GECIs are considerably slow for detecting fast electrochemical events. These drawbacks were overcome by developing genetically encoded voltage indicators (GEVIs) (Table 3). The first versions—namely, Flash [35], VSFP1 [142], and SPARC [36]—were engineered by introducing one or two FPs into the voltage-sensing domains (VSDs) of potassium or sodium channels; however, their plasma membrane targeting in neurons was found to be poor. The discovery of the non-ion channel protein *Ciona intestinalis* voltage sensor-containing phosphatase (Ci-VSP) [143] revolutionized this field as the replacement of VSDs from K2.1 potassium channel in FRET-based sensor VSFP1 with Ci-VSD resulted in the VSFP2.1 version that showed excellent subcellular localization [144]. The latter was subsequently improved; in one version (VSFP3.1), which is especially important in the context of this review, the FRET-pair was substituted with a single fluorescent domain that narrowed the optical channel occupied by the probe, facilitating multiparameter imaging [145].

The success of cpFP-based GECIs became an inspiration for attempts to engineer GEVIs that would function on the same principle with the hope that the flexible nature of a cpFP could improve the dynamic range and/or kinetic properties of the indicator. VSFP(C)cpmKate(180) and VSFP(D)cpmKate(180) were the first functional voltage probes in which cpFPs were used in their architecture [146]. In these sensors, cpmKate(180), a permuted version of mKate with a break point in one of the disorganized loops, was fused to the Ci-VSD C-terminus with a linker of varying length. The authors attempted to use other linkers and another permutation position (144) located on the beta-barrel surface; however, the fluorescence intensity of such versions was low. Both the probes were characterized by considerably poor dynamic range (approximately 1.9% and 1.2%, respectively) and kinetic constants lower than for their contemporaries (dozens of ms). Nevertheless, the half-response voltages of VSFP(C)cpmKate(180) and VSFP(D)cpmKate(180) were about −80 to −90 mV, which is closer to the physiological range, unlike that of many cpFP-based probes that were developed later. Although cpmKate(180) has a permutation point not in the close proximity to the chromophore, the authors confirmed that the cpFP-based design could be implemented in the development of GEVIs.

The authors also attempted to engineer a cpEGFP-based indicator; however, they reported that all obtained chimeras were characterized by low brightness as emission parameters of cpFPs strongly depend on the fusing partner [146]. Several years later, Barnett et al. developed such a probe and named it ElectricPk [147]. In general, it replicates the design described above with an important difference that the permutation position is located on the beta-sheet. This was achieved by screening through a library of total 90 chimeras that varied by the size of the hole on the cpFP surface and the composition of linkers. Notably, the probe performance increased by decreasing the hole size probably because of the better sensory and reporter unit coupling. Despite being the best version, ElectricPk was characterized by a very small dynamic range of −1.2%, although its kinetic properties exceeded those of other GEVIs available at that time (τ_on_ approximately 2.24 ± 0.58 ms; τ_off_ approximately 2.09 ± 0.74 ms). The authors hypothesized that this could be attributed to the fact that Ci-VSP undergoes conformational rearrangements, revealing different time scales, and ElectricPk is able to catch the fast components, unlike the probes with other types of architecture [147].

Another sensor where cpFP is fused to the C-terminus of Ci-VSD is FlicR1, which emits in the red region and is favorable for in vivo imaging [94]. That study was inspired by experimental evidence suggesting that the R-GECO1 sensing mechanism relies on the interaction of the Lys80 chain located on the beta-barrel surface with the chromophore group [122], unlike the GCaMP sensing mechanism in which the key residue is Arg from CaM [109]. Therefore, the working hypothesis was that cp-mApple from R-GECO1 represents a self-contained reporter module that could be combined with varying sensory units. The probe was engineered by inserting cpFP close to the S4 helix to enable efficient signal transition between the latter and the key lysine. Linker optimization and further random mutagenesis of the entire gene resulted in the improvement of brightness and increase in sensitivity, which was attributed to Val207 substitution. The importance of this residue was discovered accidentally, as the Val207Ala mutation led to better performance; after all possible substitutions were tested, the Val207Phe variant was found to be the best one. FlicR1 emission increases by approximately 6.6% per 100 mV; its fast kinetic components (τ_on_ approximately 3.0 ± 0.2; τ_off_ approximately 2.8 ± 0.3) are slightly greater than those of ASAP1 (mentioned below); however, their contribution to the total response was higher; therefore, both probes showed similar performance in the original study [94].

The first GEVI that combined fast kinetics and a large dynamic range was ASAP1 [86]. Crystal structures of Ci-VSP indicate that membrane potential shifts induce conformational changes in the region between the third (S3) and fourth (S4) transmembrane segments of the protein [148]. St-Pierre et al. used this information and introduced an FP into the homologous region of *Gallus gallus* VSD (Gg-VSD), containing R153Q substitution that drives Ci-VSP response to a less negative range of potentials [86]. The S3-S4 loop of Gg-VSD is shorter than that of Ci-VSD; therefore, it was suggested to ensure better conformational coupling between sensory and reporter units [86]. With the same objective, cpGFP was initially chosen because of its enhanced flexibility compared to that of intact GFP and was later replaced with cpSFGFP-OPT to increase the brightness and dynamic range. The dynamic range decreased when VSDs from *Danio rerio* or *Xenopus laevis* were tested as sensory units, and the variant with Ci-VSD showed poor plasma membrane localization. The resultant probe was named ASAP1. Its emission decreased by 17% per 100 mV, and its fast kinetic constants (τ_on_ approximately 2.1 ± 0.2 ms; τ_off_ approximately 2.0 ± 0.1 ms, contributing to 60% and 43% of response, respectively) exceeded the analogous parameters of ArcLight by 7 and 22.5 times, respectively [86]. However, the half-response voltage of the probe is about −135 mV [149], which lies relatively far from the physiological range. Further studies led to the development of improved ASAP versions. Accelerated ASAP1 kinetics apparently arises from the insertion of cpSFGFP-OPT, and the probe shows a split in both electrophysiological and optical signals, indicating the presence of an intermediate state, which is not observed in GgVSD R153Q [150]. The rational design in ASAP-Y allowed the discovery of a key substitution L158Y that eliminates the intermediate state, preserving accelerated kinetics and a large dynamic range [150]. In another study, ASAP2f was developed by linker optimization (A147S and DA148 mutations), resulting in increased response amplitude with similar temporal properties [97]. The R415Q substitution in the S4 transmembrane segment is responsible for voltage sensing and led to a probe named ASAP2s with altered kinetic constants (τ_on_ approximately 5.2; τ_off_ approximately 24.1) and significantly increased dynamic range (−39% per 100 mV) [98]. Combination of slower turn-off rate and higher response amplitude provided 90% improvement in the integral signal intensity compared to that with the initial sensor [98]. The half-voltage response for this version lies closer to the physiological range (−105 mV) [149]. Thus, ASAP1, ASAP-Y, and ASAP2f are better at resolving fast electrochemical events, whereas ASAP2s provides increased sensitivity. Finally, in a recent study, ASAP3 was developed [149] by combining mutations from ASAP2f and ASAP2s with the following random mutagenesis of the 146–151 residue region of cpFP as alterations in this fragment were suggested to affect His151 that normally stabilizes the protonated form of the chromophore. A novel protocol for multi-well library screening based on direct PCR products transfection and electroporation as a dynamic range test allowed the identification of an indicator version that showed a signal change of −50% per 100 mV. To our knowledge, this is the greatest response amplitude among all GEVIs published to date. Moreover, ASAP3 has a half-response voltage of −88 mV, which is closer to the physiological range compared to that of other family members and shows considerably rapid kinetics (τ_on_ approximately 3.7 ± 0.1 ms; τ_off_ approximately 16.0 ± 0.3 ms, constants of the fast component) [149]. 

Previously, another strategy for cpFP-based GEVI development was published [151]. The VSD-FR189–188 probe consists of cpFusionRed(189–188) and VSD from VSFP-Butterfly1.2, which serves as a linker joining native ends of the FP. VSFP-Butterfly1.2 is a FRET-based sensor with mCitrine and mKate fused to its N- and C-ends [152]. It works on the principle that shifts in the membrane potential induce conformational rearrangements in the VSD, leading to a change in the energy transfer efficiency between the FRET pair. In VSD-FR189–188, the same chain of biophysical events affects the relative positions of cpFP halves modulating the emission intensity [151]. This molecular switch, in a sense, resembles the bimolecular fluorescence complementation technique; however, in this case, rejoining of the split parts resulted not from the association of two macromolecules but from intramolecular rearrangement. VSD-FR189–188 is characterized by a small dynamic range (not more than 3%) and slow kinetics (approximately 26 ms). This probe was recently improved by linker shortening that gave 25–30-fold increase in the rate of response [153]. New versions showed responsiveness comparable to that of the best FP-based GEVIs; however, further optimization of the dynamic range is required [153]. Detailed information is listed in Table 3.

### 4.3. GEFIs for the Visualization of Oxidation and Reduction Events

Maintaining a normal redox status is essential for the functioning of cellular systems. Changes in the redox state because of stress or intrinsic cellular activity may be involved in the regulation of various cellular processes. Disruption of the redox status is associated with increased formation of reactive oxygen species (ROS)—highly reactive particles that are generally considered as products of incomplete reduction of molecular oxygen in cells. ROS can cause oxidative stress and damage cellular DNA, lipids, and proteins [154], thereby participating in the development of many pathologies such as cancer, ischemia, reperfusion injury, and some neurodegenerative diseases [155]. However, ROS may also function as signaling molecules and participate in the regulation of some physiological processes [156].

ROS are extremely reactive particles; therefore, their detection in cells is difficult. However, several ROS detection methods have been developed. For example, dichlorofluorescein derivatives that change optical parameters upon interaction with ROS are used for this purpose [157]. The disadvantages of this approach include the low selectivity of dyes and the difficulties in ROS localization in certain cellular compartments. Genetically encoded biosensors have become a kind of breakthrough in measuring ROS concentration dynamics.

The most popular GEFIs for H_2_O_2_ registration are HyPer family probes. The first such indicator, HyPer [90], was designed as a chimera consisting of cpYFP integrated into the regulatory domain of the transcription factor OxyR from *E. coli*. In the presence of H_2_O_2,_ the reduced form of OxyR is converted to its oxidized form. The key residue Cys199, which is located in the hydrophobic pocket of OxyR, reacts with H_2_O_2_ with a consequent conversion to a sulfenic acid derivative. After sulfenic acid is released from the hydrophobic pocket, it can form a disulfide bond with Cys208, causing remarkable conformational changes in OxyR. The probe shows ratiometric response with high amplitude of about 3-fold F500/f420 decrease and selectively reacts with H_2_O_2_. Subsequently, improved versions of indicators named HyPer-2 [158] and HyPer-3 [105] were developed. HyPer-2 was developed by introducing a point mutation into the OxyR domain; its dynamic range was twice that of HyPer. In addition, HyPer-2 showed slower oxidation and reduction than HyPer: both half-oxidation and half-reduction times of HyPer-2 were twice as those of HyPer. HyPer-3 was also developed by introducing a point mutation into the sensory unit of HyPer, leading to a greater dynamic range (about 6-fold) and faster kinetics of response, thereby combining the strengths of the previous versions of the biosensor. Because the fluorescent signal of HyPer is pH-dependent, its variant with Cys199 replaced for Ser (C199S), named SypHer, was developed as an appropriate pH control for using in parallel experiments. Several improved versions of SypHer such as SypHer2 [159] and SypHer3s [160] are available. SypHers are now used as pH indicators in living systems [161].

A red variant of HyPer, named HyPerRed [103], was designed by replacing cpYFP with the RFP cp-mApple. For this probe, peptide linkers between sensory and reporter units were optimized using random mutagenesis. HyPerRed is an intensiometric probe with about 2-fold amplitude response and is pH-sensitive; hence, HyPerRed-C199S should be used in parallel. Since the tissue penetration of light increases with an increase in wavelength, HyPerRed fits better for in vivo imaging. The red variant of the indicator also allows the visualization of H_2_O_2_ in multiparameter mode. Since HyPer reacts with H_2_O_2_, its possible antioxidant role is an important issue. However, in most experiments, HyPer showed no influence on the H_2_O_2_-dependent physiological processes [162]. The only exception observed is the *Arabidopsis thaliana* model [163].

For the detection of another type of ROS, organic hydroperoxides (OHPs), a genetically encoded sensor, has also been developed [164]. This probe is constructed by inserting cpVenus into conformationally mobile α-helix of the transcriptional factor OhrR that participates in controlling the OHP detoxification apparatus of bacteria. The probe shows higher selectivity to OHPs than to other types of ROS.

Methionine can be converted by biological oxidants such as ROS to the R and S diastereomers of methionine sulfoxide (MetO) and can be used as a marker of oxidative damage to proteins and metabolites. Genetically encoded fluorescent probes for R- and S-MetO have been developed [165]. These probes have a rather interesting design: the cpYFP domain is located between *S. cerevisiae* methionine sulfoxide reductase A or B (MSRs) and their specific thioredoxins (Trx1 or Trx3, respectively) with short amino acid linkers. MSRA or MSRB selectively interacts with R- or S-MetO, respectively, and a disulfide bond is formed between two redox active Cys of an MSR moiety. Next, specific Trx reduces this bond by its catalytic Cys, and a disulfide bond is formed between MSR and Trx, inducing conformational changes that cause a change in the fluorescent signal of the sensor. Both the probes show ratiometric pH-dependent response to their substrate with high dynamic range: at the optimal value of pH 7.5, MetSOx and MetROx showed about 6-fold ratio increase and decrease after oxidation, respectively. In vitro and in vivo experiments have shown that both sensors can detect changes in MetO levels from 1 to 1000 μM, which is in the physiological range of MetO. The oxidation of the sensors is reversible in cells, indicating that endogenous systems can reduce them.

Carbon monoxide (CO) plays an important role of a gasotransmitter in living systems and is involved in some pathological and physiological processes. A genetically encoded probe based on cpFP for CO detection, named COSer, has been reported [166]. This probe is based on CO-sensing heme protein CooA from *Rhodospirillum rubrum*. CooA is a transcriptional factor that consists of DNA- and CO-binding domains. The latter contains heme. The heme iron is a six-coordinate system with Pro2 as one of the axial ligands. CO displaces Pro2 and induces the twist of the long α-helix between Phe132 and Arg134, allowing the binding of CooA to DNA and activating the transcription of the *coo* operon. The sensor was designed by introducing cpVenus sequence into the F132-R134 region of the CooA α-helix. COSer shows a ratiometric response with maximal amplitude of about 2.2-fold ratio increase after CO binding and is selective for CO over other small heme ligands.

NADH, along with its oxidized form NAD^+^, is a key cofactor involved in numerous biochemical processes. The metabolic and redox states of cells depend on the [NAD^+^]:[NADH] ratio. Many genetically encoded fluorescent probes for NADH, NAD^+^, and [NAD^+^]:[NADH] ratio have been reported. All indicators are based on bacterial redox-sensing repressor Rex [167]. When the [NAD^+^]:[NADH] ratio is high, Rex forms a stable complex with DNA and one NAD^+^ molecule, thus repressing the transcription of target genes. If the NADH amount increases, it displaces NAD^+^ in Rex since the latter has considerably higher affinity to NADH than to NAD^+^. With this, Rex undergoes remarkable conformational changes and dissociates from the complex with DNA [167].

Peredox sensor, which enables measuring the [NADH]:[NAD^+^] ratio, was constructed by combining the pH-resistant FP cpT-Sapphire with *Thermus aquaticus* Rex (T-Rex) [70]. The fluorescent domain is located between two Rex subunits because the orientation of the T-Rex subunits in the dimer changes with the binding of NADH. For ratiometric signal, mCherry is fused to the C-terminus of the chimera. A version with YFP mCitrine instead of mCherry has also been reported [71]. The major limitation of Peredox is the very high affinity to the substrate, which complicates its use under physiological conditions.

Frex [99] indicators for NADH were developed on the basis of the Rex repressor from *Bacillus subtilis* (B-Rex). The design of the probes is similar to that of Peredox: the cpYFP domain is inserted between the full-length subunit and NADH-binding domain of Rex. The two best variants of the indicator obtained using single site-directed mutagenesis of the NADH-binding pocket are Frex, which responds to the analyte with a 9-fold increase in the fluorescent signal, and FrexH, which responds with a 3-fold decrease in fluorescence. These probes have differences in affinity to NADH; hence, one of them is preferred depending on the particular conditions. The main limitation of Frex family probes is their pH sensitivity; hence, a proper pH control is needed for their effective use in living systems.

RexYFP [91] is a biosensor designed based on T-Rex; however, in this construction, the cpYFP domain is integrated into the flexible loop between the DNA- and NADH-binding domains of the sensory unit. This design has an important advantage over the one described earlier: the sensor molecule is smaller and targeting it to cellular organelles is easier. The brightness and maturation at physiological temperature of the sensor were optimized using random mutagenesis. The indicator is intensiometric, and its fluorescence intensity decreases about 2-fold after NADH binding. Like many cpYFP-based indicators, RexYFP is pH-dependent, and NADPH may also affect its signal in living cells, although the indicator has considerably lower affinity for NADPH.

Another probe for [NADH]:[NAD^+^] ratio, SoNar, was constructed by inserting cpYFP into the surface loop of the NADH-binding domain of T-Rex, while the DNA-binding domain was truncated [81]. Peptide linkers of the sensor were optimized, and the probe showed extremely high amplitude of ratiometric response (about 15-fold dynamic range in vitro). The signal of SoNar did not change in response to NADPH and other nucleotides.

Based on the principle of SoNar development, researchers have developed a group of iNap indicators for [NADPH]:[NADP^+^] ratio registration [100]. Comparing the ligand-binding pockets of NADH- and NADPH-binding proteins, the authors revealed the main differences in their structural organization. The data obtained were used to design mutations that could switch the substrate specificity of SoNar to NADPH. Thus, iNap1–iNap4 sensors with similar spectral properties and affinities to NADPH of about 2.0, 6.0, 25, and 120 μM, respectively, were created. The sensors showed high dynamic range of about 900%. The variant of the sensor with completely abolished ligand-binding ability, named iNapc, is proposed to be used for pH control.

A genetically encoded probe that reports only NAD^+^ concentration, has also been developed [168]. This sensor is designed by integrating cpVenus into DNA ligase from *Enterococcus faecalis*. Point mutations were introduced in order to weaken NAD^+^ consumption and allow the monitoring of NAD^+^ within the predicted physiological range. The probe is reversible and allows ratiometric measurements with response amplitude of about 2-fold fluorescence signal decrease after NAD^+^ binding.

A reporter for ratiometric monitoring of quinones in living systems has been developed. Quinones and their derivatives participate in many important biological processes such as electron transport in cell membranes and posttranslational modification of proteins. The QSer probe was the first genetically encoded fluorescent biosensor for detecting quinones in living cells [169]. QSer was designed by inserting cpYFP between two monomeric subunits of QsrR, a transcriptional repressor from *S. aureus*. A 9° rotation occurs between the α-helices of each monomer after quinone binding, promoting cpYFP conformational change. The probe is characterized by high response amplitude (approximately 3.5-fold) and is specific to quinone molecules containing fewer than three substituted groups.

In addition to the creation of classical fluorescent biosensors, cpFP can be used to obtain biosensors with unnatural amino acids (UAAs) by using a genetic code expansion technology. The idea of obtaining such indicators is based on the introduction of UAAs into the chromophore region of a FP by coexpressing the orthogonal tRNA/synthetase pair. For example, an orthogonal tRNA/synthetase pair exists that enables the genetic encoding of *p*-azidophenylalanine in response to the amber (TAG) codon in *E. coli* and mammalian cells. UAAs chemically react with the metabolite and change the fluorescent signal. The reason for using exactly circular permutants of FP is that the chromophore of such proteins is more spatially available compared to those of native FPs. This method was used to develop sensors for the detection of hydrogen sulfide [170,171] and peroxynitrite [172]. Various redox biosensors are listed in Table 4.

### 4.4. GEFIs for Measuring Organic Metabolites

For better understanding the mechanisms of various physiological and pathological processes, determining how the metabolic fluxes of a system change is important. Different ways are available to register the changes in the concentration of a particular metabolite, such as chromatography–mass spectrometry and colorimetric and fluorometric enzyme assays [176,177]. In addition to the methods for assessing the changes in the concentration of a single metabolite, methods are available for analyzing the total metabolome. The most widely used high-throughput experiments are mass spectrometry and NMR spectroscopy-based metabolomics [178]. Although genetically encoded biosensors do not allow simultaneous analysis of numerous samples, they can be used to register the dynamics of metabolite concentrations in real time in living systems (Table 5).

Glucose is one of the main sources of energy for metabolic processes in living organisms. To date, relatively few biosensors are available that can allow the monitoring of the dynamics of glucose concentration in real time. Several FRET-based sensors, for example, series of FLIPglu probes [179,180,181,182] and glucose indicator proteins [183,184] are available. Along with these FRET-based sensors, cpFP-based sensors have also been designed.

The series of FGBP probes with differences in affinity to glucose was created by inserting cpYFP into the glucose/galactose-binding protein of *E. coli* [93]. Subsequently, the probe with physiologically relevant value of Kd, FGBP_1mM_, was selected for further characterization. FGBP_1mM_ is a ratiometric probe, and its main advantage is the high amplitude of the sensor response (up to 7-fold), which is considerably more than that for FRET-based sensors. Another glucose sensor, iGlucoSnFR [185], was designed by combining the cpGFP with a glucose/galactose-binding protein of *Thermus thermophilus.* Peptide linkers were optimized by random mutagenesis, and several point mutations were introduced in order to decrease the affinity to glucose. The probe showed intensiometric response with about 3-fold fluorescence increase after glucose binding and had Kd of 7.7 mM, which is physiologically relevant. iGlucoSnFR-TS [186], also called Sweetie-TS, was created by replacing cpGFP with a pH-stable FP cpT-Sapphire. This probe is considered as a fluorescence lifetime sensor that allows the conversion of measurements into actual concentrations in vivo. Peptide linkers between reporter and sensory units were optimized, and the probe with high fluorescence lifetime change and low pH sensitivity was selected from the library of mutants. The fluorescence lifetime change of the sensor is about 0.38 ns in HEK273T cells for a range of glucose concentrations (0.01 to 30 mM), and the signal is minimally affected by pH in the range of 7.1–7.4, making it a suitable sensor for measuring cytosolic glucose concentration.

Among the sensors for critically important cell metabolites, there are indicators that report the [ATP]:[ADP] concentration ratio [80,104]. In this case, competition between ATP and ADP for the ligand-binding center allows the sensor to report precisely the ratio of the concentrations of these two metabolites. The first such reporter, named Perceval, was designed by inserting the cpmVenus sequence into the flexible T loop of bacterial regulatory protein GlnK1 from *Methanococcus jannaschii* [80]. The original version of the sensor was optimized using semi-random mutagenesis targeting residues involved in ATP binding and T loop conformational rearrangement in order to increase the specificity, accelerate the kinetics of the response to change in [ATP]:[ATD] ratio, and improve the affinity of the instrument to be applicable in living systems. Subsequently, an improved version of the indicator, called Perceval HR, was released [104]. This sensor has significantly greater response amplitude (more than 8-fold change compared to the initial 2-fold) and can be effectively used under physiological conditions.

The cpFP-based indicators that register a change in the concentration of ATP only have also been developed [187,188]. However, several FRET-based sensors for ATP have been already reported, for example, ATeam probes, optimized for use under various conditions [60,189,190]. The major limitation of such probes is that the presence of two FPs in a sensor molecule can cause problems associated with the simultaneous maturation of two chromophores and degradation of the sensor inside the cell. Thus, malfunction of sensor molecules can occur, and their concentration may depend on the cell growth rate. These disadvantages may be overcome by generating single FP probes. The first single cpFP-based ATP indicator was the ratiometric probe QUEEN [187]. It was designed by integrating the cpEGFP sequence into the F_0_F_1_-ATP synthase epsilon subunit. Three variants of QUEEN with different affinity, one of which (QUEEN-2m) has physiologically relevant Kd value, and one is insensitive to ATP (QUEEN-NA), have been reported. Recently, a series of biosensors for ATP with different properties, named iATPSnFRs, was published [188]. The design of these probes was based on that of QUEEN with the following differences: cpEGFP was replaced with cpSFGFP, peptide linkers were optimized, and some point mutations were introduced into the sensory unit. The resulting biosensors were characterized by large ATP-dependent fluorescence intensity increase: dF/F for iATPSnFR^1.0^ is 2.4 and for iATPSnFR^1.1^ is 1.9, which is considerably more than that for QUEEN and ATeam. The probes function as single-wavelength sensors with little sensitivity to ATP metabolites.

GTP plays the role of an energy source in organisms as well as participates in cellular signaling, including the activation of G-proteins, which are signal transducers that transmit signals from various hormones, neurotransmitters, and chemokines [191]. A group of GTP sensors, named GEVALs (GTP evaluators), has recently been reported [101]. The indicators were created by inserting cpYFP into the flexible region of the FeoB G-protein domain. The sensor was further optimized by introducing mutations to weaken or eliminate GTP binding, and following versions with different substrate-binding constants were obtained: GEVAL30, GEVAL260, GEVAL530, GEVAL1150, and GEVAL2300, where the number in the sensors’ names shows K*eff* in μM. The response amplitude of all versions was about 2-fold F400/F485 increase after GTP binding. Furthermore, a pH-control version, GEVALNull, has been reported. These probes are equally selective for GTP and dGTP.

Citrate is one of the most important cell metabolites since it is the starting point of the tricarboxylic acid cycle. It participates in fatty acid biosynthesis by supplying acetyl-CoA equivalents in the cytosol as well as regulates fatty acid synthesis and glycolytic/gluconeogenetic switch by the allosteric control of the enzyme phosphofructokinase. The FRET-based biosensor for citrate detection has been previously reported [192]. Subsequently, the first citrate sensor based on cpFP was developed [92]. This sensor was designed by inserting the cpGFP domain in the *Klebsiella pneumoniae* CitA protein, a highly specific citrate receptor. Some point mutations previously described for accelerating chromophore maturation and mutations optimized for ratiometric Pericam were introduced into the cpGFP sequence. The sensor response is about 2-fold, which is almost twice than that for the previous FRET indicators.

Maltose, a disaccharide, is split into two glucose molecules in living organisms under the action of maltase enzyme. It is used in the manufacture of confectionery products, as well as in food products for children because of its low allergenicity. Genetically determined absence of maltase in humans causes maltose intolerance, requiring its elimination from the diet and supplementation with maltase. A simple cpFP-based maltose indicator [193] was designed by inserting an improved cpGFP with better brightness, spectral properties, and fluorophore maturation rate, as well as bearing the mutation S65T, into linker loop between the N- and C-terminal domains of *Thermotoga maritima* MBP. The sensor is intensiometric and shows an approximate 20% amplitude response. A family of maltose indicators has been described in another study [194]. The maltose-binding protein from *E. coli* has been chosen as a sensory domain. These sensors differ in not only affinity to the substrate but also reporter unit: sensors based on cpCFP, cpBFP, cpGFP, and cpYFP have been developed. The authors also showed that the ligand-binding specificity of the sensor can be changed from maltose to sucrose by introducing four point mutations previously shown to confer MBP with an affinity for sucrose.

A sensor for recording the dynamics of phosphonate concentrations in living systems has also been described [96]. The most abundant natural phosphonate is 2-aminoethylphophonate (2-AEP), which is a precursor in the biosynthesis of phosphonolipids, phosphonoproteins, and phosphonoglycans. Previously, a fluorescent biosensor was constructed by the covalent attachment of environmentally sensitive fluorescent dyes to introduced cysteines of EcPhnD (periplasmic-binding protein of phosphonate uptake and utilization system from *E. coli*) [195]. However, in that study, the authors relied on the structure of the EcPhnD homologue, sulfate-binding protein, although it shares only 37% sequence identity with PhnD. Subsequently, the crystal structure of EcPhnD was determined, and a biosensor designed by integrating cpGFP in the EcPhnD sequence was developed [96]. This sensor has been optimized by screening of linker mutants. Interestingly, the variant with the deletion of two amino acid residues of the sensory domain right before the cpGFP sequence, obtained by linker mutagenesis, was selected. The sensor has 2.6-fold amplitude response to 2-AEP and was suitable for the screening of phosphonates in living systems.

Histidine is an essential amino acid and a precursor for histamine; it is of vital importance for neurotransmission and neuromodulation [196]. The FRET-based biosensor for histidine detection based on bacterial periplasmic-binding protein (PBP) HisJ, which specifically binds to histidine, and its analogue with circularly permuted HisJ subunit, have been reported [197]. Subsequently, a single FP-based sensor, named FHisJ, was designed by integrating the cpYFP domain into the flexible regions of HisJ [95]. This indicator has a high amplitude response (520%), which is about 8-fold higher than that for the FRET permuted biosensor, and the value of its dissociation constant is 22 μM, which is physiologically relevant. Detailed information is listed in Table 5.

### 4.5. GEFIs for Cellular Signaling Visualization and Neurotransmitter Measurement

One of the most important properties of living cells is their ability to adapt to environmental perturbations. In order to maintain their metabolism adjusted for current needs, cells have developed complicated signaling systems in the course of evolution [198]. These systems provide metabolic switches that allow either metabolism buffering or shifting depending on the precise situation with different degrees of biochemical pathways rearrangements and varying temporal dynamics. The fastest events include manipulating with ion concentrations and proceed at millisecond rates, whereas the slowest rely on affecting gene activity and require hours to be completed. This area of investigation is essential in modern research as deciphering signaling intercalations is of remarkable importance for understanding development, metabolic diseases, tumorigenesis, nervous system functioning, and consequent drug development. Hence, the real-time kinase activity dynamics, G-protein-coupled receptor activation, secondary messengers, and neurotransmitter concentration shifts at single cell resolution have been visualized by using genetically encoded indicators. To date, numerous indicators of varying colors have been developed, and some of them involve the use of cpFPs (Tables 6–8).

Secondary messengers from inositol phosphate family orchestrate signal transduction in many pathways that are involved in cell differentiation, proliferation, neural plasticity, and muscle contraction [199]. They are often recognized by pleckstrin homology (PH) domains; however, the latter often lack conformational mobility sufficient for FRET-based probe development. Inspired by successful engineering of a split phospholipase Cδ1 PH domain that retained affinity and selectivity for the natural ligand [200], Sakaguchi et al. developed a genetically encoded probe for Ins(1,3,4,5)P_4_ by inserting cpGFP into a mobile loop of the PH domain from Bruton’s tyrosine kinase [67]. In this probe, the flexible nature of cpFP provides efficient conformational coupling of sensory and reporter units, converting small protein rearrangements into an optically detectable signal. The authors speculated that ligand binding disrupts His148 interaction with the chromophore that shifts the acid-base equilibrium toward the deprotonated form. This results in a 1.5-fold ratiometric response.

Another important secondary messenger related to phospholipids is diacylglycerol (DAG). It is mostly generated in the course of phospholipase C reaction and serves as a key inducer of protein kinase C (PKC) [201]. Given that the activation of the latter usually requires Ca^2+^ influx, GECIs have been successfully implemented for real-time imaging of this signaling pathway. However, this approach has a significant drawback coming from the fact that Ca^2+^ ions play a role in other biochemical processes. The need for clearly separating these factors led to the development of cpGFP-based DAG probes [202]. They utilize conformational rearrangements occurring in PKCδ under ligand binding, namely, the loss of the contact between enzyme and pseudosubstrate domains of the protein. This isoform was selected because experimental evidence suggests that its C2-domain is insensitive to Ca^2+^ [203], and the C1-domain shows high affinity for analyte [204]. Among 30 engineered variants, Tewson et al. selected Upward and Downward DAG sensors that differed by the direction of the response, which was about 40% in HEK293 cells for both the probes. This response magnitude was higher than that for the contemporaries [202]. Subsequent optimization led to the development of Upward and Downward DAG2 versions with improved dynamic range [26]. Moreover, the first prototypes based on cpmNeon, a brighter protein were reported [205]. Interestingly, Upward and Downward DAG2 probes showed different response kinetics: return to the baseline was faster for the Upward DAG2 probe [26]. The authors suggested that this might be because of photobleaching; therefore, intensiometric probes with different directions of response can be good controls for each other. Notably, other designs for DAG sensors have been reported. One type is based on the translocation of activated PKC-FP chimeras to the membranes [206] and another is FRET-based [207]. Translocation readout is often not sufficiently quantitative and requires high-resolution imaging for good results, whereas FRET-based probes show low maximal amplitude.

Many biochemical events are induced by cyclic nucleotides cAMP and cGMP generated by adenylate cyclase and guanylate cyclase, respectively. The drawbacks of the already available FRET-based biosensors for cGMP visualization were overcome by Nausch et al., who engineered cpEGFP-based probes by inserting the latter into the regulatory domains of protein kinases G (PKG) I α or β [87]. This resulted in the development of three indicators, namely, α-, β-, and δ-FlincGs, that differed by affinity, cGMP/cAMP selectivity, and dynamic ranges. δ-FlincG was obtained from the α-version by removing the entire PKGIα N-terminal domain, and it showed a 3.5-fold ratiometric excitation response, unlike the other variants that responded intensiometrically despite having both 410 nm and 480 nm excitation maxima in their spectra. Moreover, the responses of α- and β-FlincGs were reduced during cell imaging compared to those obtained in vitro; the authors hypothesized that it can be explained in the terms that unlike the δ-version, the α- and β-FlincGs contain dimerization interfaces driving them to interact with cellular PKGs. The affinity of δ-FlincG to cGMP was shown to be similar to the wild-type protein; therefore, it cannot likely act as a metabolic sink, and its pKa was estimated to be 6.1, making the probe relatively resistant to pH shifts in the physiological range. However, subsequent studies on the issue [208] questioned these data and provided a different pKa value of 7.5; moreover, the authors showed that the alteration of the C-terminus remarkably affects the performance of the sensor, in contrast to that mentioned in the previous study [87]. Further optimization led to the development of FlincG2 and FlincG3 [208]. The former retains the δ-FlincG sequence except for the C-terminal part, whereas the latter has M335K substitution in the cpFP and N-terminal His-tag that was shown to increase brightness. The affinity of FlincG3 to cGMP was lower than that of the δ-version, which might be preferable in some situations, but the ratiometric response characteristic was lost. Detailed information is listed in Table 6.

The first published cpFP-based probe for cAMP measurement was cADDis [205]. It was engineered in order to develop a sensor with a good signal-to-noise ratio for multi-well plate automatic assays. The crystal structure of guanosine exchange factor EPAC2 revealed that ligand binding induces twisting in a hinge that connects the catalytic and regulatory regions [210]. While testing prototypes varying by cpGFP insertion position inside this sequence and linker composition a chimera with a dynamic range of about 35% [205] was found that became the final version. Further characterization revealed that cADDis shows optimal sensitivity at analyte concentrations between 10 and 100 μM.

Another green cAMP probe based on cpFP was recently published [79]. cAMPr was developed by connecting the catalytic and regulatory Iα subunits of protein kinase A with cpGFP as a linker. The authors suggested that, in the ligand-free state, the subunits would be connected to each other, and relaxation induced by cAMP would be transmitted to the reporter domain affecting its fluorescence. Membrane localization and interaction with wild-type enzyme was avoided by removing the dimerization/docking domain from the regulatory subunit. Next, the second cAMP-binding site was deleted to decrease the affinity of the probe, expanding its field of application as cAMP concentration can vary in a wide range. Further linker optimization led to the development of the final version with a dynamic range of about 50%. 

Ohta et al. focused on the high affinity and selectivity of PKA RIα and engineered a red cAMP probe named R-FlincA [209]. In this case, the architecture of the sensor was different as cp146mApple was directly inserted into the high-affinity cAMP-binding motif of this protein. To our knowledge, R-FlincA is characterized by a very large dynamic range of 860%, which is the highest among cAMP probes, and the best cAMP/cGMP selectivity. Its dissociation constant for analyte is about 0.3 µM, which surpasses the corresponding values for Flamindo2 and Pink-Flamindo (3.2 µM and 7.2 µM, respectively) [209]. The main drawbacks of this probe are pH sensitivity and low brightness.

Many signaling pathways rely on the activation of protein kinases (PKs) that are capable of phosphorylating target molecules affecting their functions. Different classes of enzymes and transcriptional factors are subjected to such regulation. To date, numerous genetically encoded probes have been used for visualizing PK activity; however, most of them utilize FRET technology, and only few are based on cpFPs [211]. This design based on cpFPs was first implemented in sinphoses, which represent a family of probes for insulin-induced protein phosphorylation [212]. These probes were constructed by joining the SH2n domain from the p85 regulatory subunit of phosphatidylinositol 3-kinase and Y941 domain from IRS-1 with cpECFP, cpEGFP, or cpCitrine as linkers. Efficient interaction with insulin receptor was confirmed by fusing a PH domain and a phosphotyrosine-binding (PTB) domain derived from IRS-1 to the N-terminus of the probes. When insulin receptor is activated, it phosphorylates Y941, converting it to a binding partner for SH2n with subsequent spatial reorganization of the sensor that can be detected by cpFPs. Sinphoses are characterized by maximal amplitudes of about 10–15%, with the cyan version being the only one relatively resistant to pH shifts. Recently, the color options for cpFP-based PK sensors have been complemented by ExRai family members that utilize cpGFP as a reporter unit [28]. Basically, these probes have the same design, in which the phosphoaminoacid-binding domain is represented by FHA1 from AKAR, and PKA, PKB, or PKC substrate serves as the phosphate acceptor moiety, generating ExRai-AKAR, ExRai-AtkAR, and ExRai-CKAR, respectively. Unlike GCaMP3 that contains the same reporter unit, all the above probes showed ratiometric excitation response, indicating that in ExRais the wtGFP chromophore behavior is ‘rescued’ by fusion partners. ExRai-AtkAR and ExRai-CKAR were characterized by the greatest dynamic range among other genetically encoded probes for the same biochemical events. The maximal response of ExRai-AKAR was more than 2-fold, that is, 3- and 1.7-times higher than that of AKAR4 and AKAR3ev, respectively [28]. The authors also showed that the ExRai family is color-tunable by converting ExRai-AKAR and ExRai-CKAR to blue-shifted versions by substituting cpGFP for cpCFP or cp-T-sapphire. Detailed information is listed in Table 7.

One more field of interest is to register the real-time dynamics of neurotransmitter release in neuronal tissue. Such data remarkably contribute to our understanding of neurogenerative diseases, physiological regulation, and behavior and facilitate rational drug development. Traditional approaches to investigate this issue are microdialysis, current measurement by using a microelectrode, and enzyme coupling. Despite their contribution, to our knowledge, these approaches lack the desired spatial and temporal resolution; genetically encoded biosensors can overcome these obstacles. Thus, iGluSnFR, a cpFP-based probe for glutamate, which is one of the most widespread neurotransmitters both in vertebrates and invertebrates, was developed [84]. The authors had previously engineered sensors by using periplasmic bacterial proteins (PBPs) as sensory units—namely, *E. coli* maltose-binding protein MalE [194] and *E. coli* phosphonate-binding protein PhnD [96] (discussed before)—and implemented this technique to *E. coli* GltI protein. The cpGFP insertion points were selected on the basis of global structural similarity of a homologous protein from *Shigella flexneri* to the resolved structure of MalE. Primer optimization resulted in the final version that showed approximately 450% response under glutamate binding. Finally, IgG kappa secretion tag, Myc tag, and platelet-derived growth factor receptor domain were added for desired subcellular targeting. iGluSnFR has high affinity and is selective with the exception for aspartate. cpGFP was substituted with cpSFGFP, and a version with improved brightness, named SF-iGluSnFR, was developed [102]. Additional modifications that affect the kinetic properties of the probe were discovered. Considering that mutations of residues in the ‘hinge’ of PBPs can allosterically alter affinity, a version with A184S substitution was engineered. It showed a lower dissociation constant and slower off-rate, thereby allowing better integral signal collection. S72A afforded the opposite effect that might be beneficial for large synapse imaging where glutamate clearance is limited. By introducing mutations to the reporter unit, the authors developed SF-Azurite-iGluSnFR and SF-Venus-iGluSnFR with different spectral properties (linker optimization was required for the former). Finally, in another study, a red version—R-iGluSnFR1—was presented [58]. cpmApple was chosen as a reporter unit for the same reason as in the case of FlicR1 [94] (see before). After linker optimization and seven rounds of random mutagenesis, the authors obtained the final version with a dynamic range of 4.9-fold. Its pKa values were 5.1 and 8.3 for free and bounded configurations, respectively, indicating that a molecular switch may consist in the glutamate-induced conformational rearrangement, preventing the stabilization of the deprotonated state by the key lysine residue. Unlike the original version, R-iGluSnFR1 is sensitive to not only glutamate and aspartate, but also asparagine. In the same study, both sensors were converted to non-permutated forms bearing Camgaroo-like topology [58].

Another probe based on PBP is iGABASnFR; it can allow GABA monitoring [85]. Initially, the authors attempted to utilize the well-described Atu2422 and Atu4243 proteins from *Agrobacterium tumefaciens* as sensory units; however, the former lacked selectivity and affinity for the target analyte, and the latter failed to provide good membrane localization. Next, a protein homologous to Atu4243 from *Pseudomonas fluorescens* was detected using genome screening; insertion points for cpSFGFP were selected on the basis of structural similarity. F101L substitution was introduced into the hinge region to allosterically modulate affinity, and another mutation N260A significantly increased the brightness in HEK cells possibly because of the potential interaction of this residue with cpFP or its capacity to alter the glycosylation site. Linker re-optimization and F145W substitution in cpSFGFP led to the final version with high selectivity, sensitivity, and a dynamic range of 250%. Other mutations, namely, F102G and 102Y.Y137L combination, provided lower dissociation constant and increased maximal response amplitude of up to 450%.

Lastly, cpFP-based probes utilizing G-protein coupled receptors (GPCRs) as sensory modules are described. All these probes share topological similarity in cpFP insertion position located in the internal loop 3 region that in β-2 adrenergic receptor undergoes significant conformational rearrangement under ligand binding that might provide a molecular switch for sensor development [213,214,215,216]. A family of dLight probes for dopamine visualization was engineered by introducing cpGFP into dopamine receptors D1, D2, or D4 at different positions with six resulting variants, covering a broad spectrum of affinity (dissociation constants from 4.1 nM to 2.3 µM) and dynamic ranges (from 170% to 930%) [82]. Although dLight1.1 and dLight1.2 are optimized variants combining a balance between response amplitude and affinity, the authors claimed that other versions are suitable for specific purposes, for example, dLight1.3 and dLight1.4 are useful for measuring synaptic release and tonic dopamine transients, respectively. Such sensor design is applicable to other GPCRs that was shown by developing a range of probes based on β-1 and β-2 adrenergic receptors, κ- and μ-type opioid receptors, α-2 adrenergic receptors, 5-hydroxytryptamine (serotonin) receptor-2A, and melatonin type-2 receptor, but without further optimization [82].

Another family of cpFP-based probes for dopamine visualization is GRAB_DA_ [88]. These probes were developed independently from dLight sensors on the basis of the same principles mentioned above. Each human DA receptor subtype was tested, and DRD2-cpEGFP chimera was selected as it provided good membrane trafficking and showed high affinity for analyte. After additional mutations were introduced, two versions were selected that differed in their dissociation constants (130 nM and 10 nM for GRAB_DAm_ and GRAB_DAh_, respectively) and shared a dynamic range of about 90%, which is smaller than the corresponding value for dLights. However, compared to the latter, GRAB_DA_ sensors were optimized for brightness [88]. Both GRAB_DA_ and dLights are selective with the exception for norepinephrine; however, considering the difference in affinities and physiological concentrations of dopamine and norepinephrine, this feature is not a limitation for their in vivo implementation. As FRET-based dopamine probes are characterized by a dynamic range of not more than 10%, both sensor families represent a remarkable breakthrough in this field. The spatial structure of DRD2 receptor has been resolved recently, and this might provide useful information for the rational optimization of these indicators [217].

GACh probes for acetylcholine measurements were engineered on the same principle [83]. Human muscarinic acetylcholine receptors 1–5 were tested as candidates for sensory unit with their internal loop 3 substituted with modified homologous region from β-2 adrenergic receptor. This was required because the initial length of this sequence in muscarinic acetylcholine receptors is very large; the authors intended to avoid the possible problems associated with expression and trafficking resulting from a lengthy cpGFP-containing loop. M_3_R chimera showed excellent membrane targeting and could respond to the analyte when expressed in HEK293T cells with amplitude of 30%. Linker optimization allowed to increase the dynamic range of the probe to 90%.

Finally, a family of norepinephrine-sensing cpFP-based probes, named GRAB_NE_, was developed [89]. Among all tested candidates for the sensory unit, α-2 adrenergic receptor showed the best performance. Two versions—namely, GRAB_NEm_ and GRAB_NEh_—differing by the presence of a mutation T6.34K in a region close to a conservative site E6.30 were developed; this mutation lowers the dissociation constant of the latter. Thus, the maximal response amplitudes and dissociation constants are 230% and 930 nM for GRAB_NEm_ and 130% and 100 nM for GRAB_NEh_, respectively. Both the probes could not distinguish between epinephrine and norepinephrine; however, the authors claimed that it was not a concern in mammalian central nervous tissue as most human adrenergic receptors also respond non-discriminately to both ligands [89]. Therefore, GRAB_NE_ probes reveal the regions where noradrenergic or adrenergic signaling occurs.

Notably, none of the cpFP- and GPCR-based probes disrupted the normal signaling pathways, which is experimentally proven. The theoretical basis underlying this observation is that internal loop 3 region is crucial for G-protein/arrestin and GPCR interactions, and a bulky moiety of cpFP hinders such coupling [191,218]. In addition to the approaches for neurotransmitter visualization already mentioned above, several other methods exist that utilize fluorescent reporters for either direct analyte detection or GCPRs activation. In some cases, cpFP-based probes provide several advantages over such reporters: FRET-based sensors usually do not have a large dynamic range, CNiFERs [219] technology is invasive and lacks spatial resolution, and TANGO assay [220] requires long time for signal amplification. Detailed information is listed in Table 8.

## 5. Conclusions

The direction of creating biosensors based on cpFP will continue to evolve. This approach provides researchers with an unlimited field for creative activity. Developing a new biosensor requires the selection of a sensory domain, which interacts with the ligand of interest or a defined cellular parameter. The diversity of proteins in various organisms allows the creation of a sensor for any component. However, for successful implementation, detailed knowledge of the mechanisms of protein functioning and their structures is necessary.

Moreover, improving the already existing biosensors is necessary. Many of them are not sufficiently bright to be used in complex in vivo systems. Some have low response amplitude. Some examples of biosensor families clearly demonstrate that many characteristics of a biosensor can be significantly improved even on the basis of the same cpFP. Future studies need to be directed toward development of new cpFPs that have improved characteristics, in particular, brightness and fluorescence signal stability across physiological pH changes. Studies need to also focus on expanding the color range of the existing biosensors, which would allow combining probes within the same system for more informative multiparameter imaging.

## Figures and Tables

**Table 1:** Calcium GEFIs.

Name	Permutant	Sensor Unit	λex, nm	λem, nm	Kd	RA	Reference
B-GECO1	Y66H-mutated G-GECO	**CaM**-M13	378	446	164 nM	7-fold	[119]
BCaMP1c	cpEBFP	**CaM**-M13	350	460	599 nM	2-fold	[122]
CyCaMP1a	cpECFP	**CaM**-M13	450	510	421 nM	2.6-fold	[122]
MatryoshCaMP6s	cpEGFP_LSSmOrange	**CaM**-M13	440/485	510/570	197 nM	8.5-fold	[73]
sfMatryoshCaMP6s	cpEGFP_LSSmOrange	**CaM**-M13	440/485	510/571	501 nM	7.6-fold	[73]
sfMatryoshCaMP6s-T78H	cpEGFP_LSSmOrange	**CaM**-M13	440/486	510/572	270 nM	11.9-fold	[73]
GCamP3	cpEGFP	**CaM**-M13	399/496	513	542 nM	12.3-fold	[119]
GCaMP5G	cpEGFP	**CaM**-M13	430	535	450–460 nM	32.7-fold	[114]
GCaMP5K	cpEGFP	**CaM**-M13	NM	NM	189 nM	9.4-fold	[114]
G-CaMP6	cpEGFP	**CaM**-M13	400/490	515	158 nM	11.4-fold	[113]
G-CaMP8	cpEGFP	**CaM**-M13	400/490	515	200 nM	38-fold	[113]
GCaMP6f	cpEGFP	**CaM**-M13	497	515	375 nM	51.8-fold	[115]
GCamP6m	cpEGFP	**CaM**-M13	497	515	167 nM	38.1-fold	[115]
GCaMP6s	cpEGFP	**CaM**-M13	497	515	144 nM	63.2-fold	[115]
GCaMP-HS	cpEGFP	**CaM**-M13	488	509	102 nM	4.1-fold	[126]
GEX-GECO1	cpEGFP	**CaM**-M13	390/482	506	318 nM	26-fold	[119]
GEM-GECO1	cpEGFP	**CaM**-M13	390	455/510	340 nM	110-fold	[119]
G-GECO1.1	cpEGFP	**CaM**-M13	398/496	512	618 nM	26-fold	[119]
CEPIA	cpEGFP	**CaM(cfGCaMP2)**-M13	488	512	368 μM	4.2-fold	[118]
G-CEPIA1er	cpEGFP	**CaM (cfGCaMP2)**-M13	488	512	672 μM	4.7-fold	[118]
GEM-CEPIA1er	cpEGFP	**CaM**-M13	395	460/510	558 μM	21.7-fold	[118]
GR-GECO1.2 (green)	cpMaple	**CaM**-M13	383/488	506	74 nM	3.9-fold	[121]
Flash-pericam	cpEYFP	**CaM**-M13	410/494	514	0.7 μM	8-fold	[66]
Ratiometric-pericam	cpEYFP	**CaM**-M13	415/494	517	1.7 μM	10-fold	[66]
Inverse-pericam	cpEYFP	**CaM**-M13	490	513	0.2 μM	5-fold	[66]
Case12/16	cpYFP	**CaM**-M13	491	516	1 μM	12/16-fold	[127]
YCaMP1b	cpEYFP	**CaM**-M13	500	520	809 nM	9.2-fold	[122]
R-GECO1	cpmApple	**CaM**-M13	445/561	589	142 nM	16-fold	[119]
CH-GECO2.1	cpmCherry	**CaM**-M13	588	604	6 nM	2.5-fold	[128]
R-GECO2L	cpmApple	ckkap-WL-Calmodulin	575	600	26 nM	4.1-fold	[120]
RCaMP1h	cpmRuby	**CaM**-M13	571	594	1.3 μM	10.5-fold	[122]
O-GECO1	cpmApple	**CaM**-M13	420/543	564	1.5 μM	146-fold	[129]
R-GECO1.2	cpmApple	**CaM**-M13	420/556	585	1.2 μM	33-fold	[129]
CAR-GECO1	cpmApple	**CaM**-M13	560	609	490 nM	27-fold	[129]
R-CEPIA1er	cpmApple	**CaM (cfGCaMP2)**-M13	561	584	565 μM	8.8-fold	[118]
REX-GECO1	cpmApple	**CaM**-M13	480	585	240 nM	100-fold	[130]
LAR-GECO1	cpmApple	**CaM**-M13	574	598	24 μM	10-fold	[131]
GR-GECO1.2 (red)	cpMaple	**CaM**-M13	558	582	90 nM	6.7-fold	[121]
K-GECO1	cpFusionRed	ckkap-Calmodulin	565	590	165 nM	12-fold	[132]

The raw colors refer to the colors of fluorescent proteins used as reporter domains. Kd—dissociation constant. NM—not measured. RA—response amplitude.

**Table 2:** GEFIs for inorganic ions measuring.

Name	Analyte	Permutant	Sensor Unit	λex, nm	λem, nm	Kd	RA	Reference
ZnGreen2	Zn^2+^	cpmTFP	Zinc hook peptide from *Pyrococcus furiosus* Rad50	462	492	20 mM	8.7-fold	[139]
Am Tryoshka 1;3 LS-F138I	NH^4+^	cpsfGFP, LSSmOrange	AtAMT1;3	440/480	510/570	84.5 μM	−15–22% green channel	[73]
Am Tryoshka 1;3 LS-F138I-T78H	NH^4+^	cpsfGFP, LSSmOrange	AtAMT1;3	440/480	510/570	64.4 μM	−30% green channel	[73]
GCS-2	Cu^2+^	cpGFP	ATCUN motif (Gly-Gly-His)	480	510	8 nM	85%	[135]
GZnP1	Zn^2+^	cpGFP	2 zinc fingers of the yeast transcription factor Zap1 (ZF1 and ZF2)	496	512	NM	15%	[138]

The raw colors refer to the colors of fluorescent proteins used as reporter domains. Kd—dissociation constant. NM—not measured. RA—response amplitude.

**Table 3:** Voltage GEFIs

Name	Permutant	Sensor Unit	λex, nm	λem, nm	RA	Kinetic Range	Reference
ElectricPK	cpEGFP	CiVSD	NM	NM	−1.2% per 100 mV	In ms range	[147]
ASAP1	cpsfGFP-OPT	GgVSD	NM	NM	−18% per 100 mV	In ms range	[86]
ASAP2f	cpsfGFP-OPT	GgVSD	NM	NM	−35% per 100 mV [149]	In ms range	[97]
ASAP2s	cpsfGFP-OPT	GgVSD	NM	NM	−39% per 100 mV	In ms range	[98]
ASAP-Y	cpsfGFP-OPT	GgVSD	NM	NM	Improved kinetics due to elimination of intermediate state		[150]
ASAP3	cpsfGFP-OPT	GgVSD	NM	NM	−51% per 100 mV	In ms range	[149]
VSFP(D)cpmKate(180)	cpmKate	CiVSD	NM	625	1.2% per 200 mV	Slow kinetics	[146]
VSFP(C)cpmKate(180)	cpmKate	CiVSD	NM	625	1.9% per 200 mV	Slow kinetics	[146]
FlicR1	cpmApple	CiVSD	570	597	6.6% per 100 mV	In ms range	[94]
VSD-FR189–188	cpFusionRed	Kv3.1-CiVSP	574	610	3% per 100 mV	Slow kinetics	[151]

The raw colors refer to the colors of fluorescent proteins used as reporter domains. NM—not measured. RA—response amplitude.

**Table 4:** Redox GEFIs.

Name	Analyte	Permutant	Sensor Unit	λex,nm	λem,nm	Kd	RA	Reference
Peredox	NADH/NAD^+^	cpT-Sapphire	T-Rex	400/587	510/610	<5 nM for NADH for the first version P0	2.5-fold	[70]
cpGFP-Tyr66pAzF	H_2_S	cpGFP	Modified chromophore: UAAs are introduced	490	520	NM	≈ 3.5-fold	[170]
hsGFP	H_2_S	cpsfGFP	Modified chromophore: UAAs are introduced	454	500	H_2_S detection limit = 435 nM	23-fold	[171]
pnGFP	ONOO^-^	cpsfGFP	Modified chromophore: UAAs are introduced	484	508	ONOO^-^ detection limit = 553 nM	≈ 25-fold	[172]
NeonOxIrr	H_2_O_2_	cpmNeonGreen	OxyR	508	520	higher and similar sensitivity to low H_2_O_2_ concentra-tions, compared with HyPer-3	2.8-fold	[173]
TriPer	H_2_O_2_	cpYFP	OxyR	405/488	NM	NM	NM	[174]
HyPer	H_2_O_2_	cpYFP	OxyR	420/500	516	25 – 250 nM H_2_O_2_ detection (in vitro)	3-fold	[90]
HyPer-2	H_2_O_2_	cpYFP	OxyR	420/500	516	The same as for HyPer	6-fold	[158]
HyPer-3	H_2_O_2_	cpYFP	OxyR	420/500	516	The same as for HyPer	6-fold	[105]
Frex	NADH	cpYFP	B-Rex	420/500	518	3.7 μM	9-fold	[99]
FrexH	NADH	cpYFP	B-Rex	420/500	518	40 nM	3-fold	[99]
RexYFP	NADH/NAD^+^	cpYFP	T-Rex	490	516	180 nM	2-fold	[91]
SoNar	NADH/NAD^+^	cpYFP	T-Rex	420/485	528	0.2 μM for NADH 5.0 μM for NAD^+^	15-fold	[81]
NAD^+^ sensor	NAD^+^	cpVenus	DNA ligase	405/488	≈520	65 μM	2-fold	[168]
iNap1	NADPH	cpYFP	T-Rex	420/500	515	2.0 μM	10-fold	[100]
iNap2	NADPH	cpYFP	T-Rex	420/500	515	6.0 μM	10-fold	[100]
iNap3	NADPH	cpYFP	T-Rex	420/500	515	25 μM	10-fold	[100]
iNap4	NADPH	cpYFP	T-Rex	420/500	515	120 μM	10-fold	[100]
Qser	quinones	cpYFP	QsrR	427/496	515	NM	3.5-fold	[169]
MetSOx	S-MetO	cpYFP	MSRA and Trx1	425/505	510-516	K_0.5_ = 0.5 μM (in vitro)	6-fold	[165]
MetROx	MetO	cpYFP	MSRB and Trx3	410/500	510-516	K_0.5_ = 177 μM (in vitro)	6-fold	[165]
OHSer	ROOH	cpVenus	*Xc*OhrR	519	526	NM	2-fold	[164]
COSer	CO	cpVenus	CooA	516	528	2 μM	2-fold	[166]
HyPerRed	H_2_O_2_	cpmApple	OxyR	575	605	20 – 300 nM H_2_O_2_ detection (in vitro)	2-fold	[103]
rxRFP	general redox state	cpmApple	Redox sensitive peptides	576	600	NM	4-fold	[175]

The raw colors refer to the colors of fluorescent proteins used as reporter domains. Kd—dissociation constant. NM—not measured. RA—response amplitude. UAA—unnatural amino acids.

**Table 5:** GEFIs for organic metabolites measuring.

Name	Analyte	Permutant	Sensor Unit	λex,nm	λem,nm	Kd	RA	Reference
MBP165-cpAzurite	Maltose	cpAzurite	MBP	NM	440/480	3.3 μM	1.5-fold	[194]
MBP165-cpCFP	Maltose	cpCFP	MBP	NM	500	13 μM	1.75-fold	[194]
iGlucoSnFR	Glucose	cpGFP	*Tt*GBP	488	520	7.7 mM	3.3-fold	[185]
iGlucoSnFR-mRuby2	Glucose	cpGFP	*Tt*GBP	488/561	520/600	2.2 mM	2.0-fold	[185]
iGlucoSnFR-TS (Sweetie TS)	Glucose	cpT-Sapphire	*Tt*GBP	400/480	510	FLIM-probe		[186]
CF98	Citrate	cpGFP	CitA	413/504	518	0.1–50 mM citrate detection	2-fold	[92]
N-MBP:cGFP:C-MBP	Maltose	cpGFP	*Tm*MBP	≈490	NM	6.89 mM	1.2-fold	[193]
MBP165-cpGFP	Maltose	cpGFP	MBP	NM	≈510	4.5 μM	2.5-fold	[194]
EcPhnD90-cpGFP. L1ADΔΔ.L297R,L301R	2-AEP	cpGFP	PhnD	NM	NM	37±7 μM	2.6-fold	[96]
QUEEN-7μ	ATP	cpGFP	ε-F_0_F_1_	400/494	513	7 μM	NM	[187]
QUEEN-2m	ATP	cpGFP	ε-F_0_F_1_	400/494	513	4.5 mM	NM	[187]
iATPSnFR^1.0^	ATP	cpSFGFP	ε-F_0_F_1_	490	512	120 μM	3.4-fold	[188]
iATPSnFR^1.1^	ATP	cpSFGFP	ε-F_0_F_1_	490	512	50 μM	2.9-fold	[188]
mRuby- iATPSnFR^1.0^	ATP	cpSFGFP	ε-F_0_F_1_	490/NM	512/NM	The same as for iATPSnFR^1.0^		[188]
MBP165-cpYFP	Maltose	cpYFP	MBP	NM	525	3.3 μM	3-fold	[194]
FGBP_1mM_	Glucose	cpYFP	GGBP	420/500	515	1 mM	7-fold	[93]
GEVALs	GTP	cpYFP	FeoB	≈400/485	NM	30 μM, 260 μM, 530 μM, 1150 μM, 2300 μM	2-fold	[101]
FHisJ	Histidine	cpYFP	HisJ	420/500	515	22 μM	6.2-fold	[95]
Perceval	[ATP]:[ADP]	cpVenus	*Mj*GlnK1	405/490	520	K_R_ = 0.5	2-fold	[80]
PercevalHR	[ATP]:[ADP]	cpVenus	*Mj*GlnK1	420/500	520	K_R_ = 3.5	> 8-fold	[104]

The raw colors refer to the colors of fluorescent proteins used as reporter domains. Kd—dissociation constant. NM—not measured. RA—response amplitude.

**Table 6:** GEFIs for secondary messengers measuring.

Name	Analyte	Permutant	Sensor Unit	λex,nm	λem, nm	Kd	RA	Reference
Upward DAG	DAG	cpGFP	PKCδ without C2 domen	NM	NM	NM	45% (in vivo)	[202]
Downward DAG	DAG	cpGFP	PKCδ without C2 domen	NM	NM	NM	−40% (in vivo)	[202]
Upward DAG2	DAG	cpGFP	PKCδ without C2 domen	NM	NM	Improved version of Upward DAG		[26]
Downward DAG2	DAG	cpGFP	PKCδ without C2 domen	NM	NM	Improved version of Downward DAG		[26]
cADDis	cAMP	cpGFP	EPAC2	NM	NM	10–100 μM cAMP detection	−35% (in vivo)	[205]
cAMPr	cAMP	cpGFP	PKA-catalytic and PKA regulatory subunit Iα (RIα)	490	520	NM	1.5-fold	[79]
α-FlincG	cGMP	cpEGFP	Regulatory domain PKG Iα	480	510	35 nM	1.55-fold	[87]
β-FlincG	cGMP	cpEGFP	Regulatory domain PKG Iβ	480	510	1.1 μM	2.05-fold	[87]
δ-FlincG	cGMP	cpEGFP	Regulatory domain PKG Iα	410/480	510	490 nM	3.5-fold	[87]
FlincG2	cGMP	cpEGFP	Regulatory domain PKG Iα	480	510	Modified version of δ-FlincG		[208]
FlincG3	cGMP	cpEGFP	Regulatory domain PKG Iα	480	510	Modified version of δ-FlincG		[208]
Btk-cpGFP	inositol-1,3,4,5-tetrakisphosphate	cpGFP	PH domain from Btk	396/470	508	100 nM	1.5-fold	[67]
R-FlincA	cAMP	cp146mApple	high-affinity cAMP-binding motif from the human PKA regulatory subunit Iα (RIα)	571	590	0.3 μM	860%	[209]

The raw colors refer to the colors of fluorescent proteins used as reporter domains. Kd—dissociation constant. NM – not measured. RA—response amplitude.

**Table 7:** GEFIs for measuring kinase activity.

Name	Analyte	Permutant	Sensor Unit	λex, nm	λem, nm	RA	Reference
blueAKAR	PKA activity	cpBFP	PKA substrate/ FHA1 from AKAR	385	450	−21% (in vivo)	[28]
blueCKAR	PKC activity	cpBFP	PKC substrate/FHA1 from AKAR	385	450	−23% (in vivo)	[28]
cyan-sinphos	Insulin induced protein phosphorylation	cpECFP	PH-PTB/SH2n	NM	482	−10% (in vivo)	[212]
green-sinphos	Insulin induced protein phosphorylation	cpEGFP	PH-PTB/SH2n	NM	515	15% (in vivo)	[212]
ExRai-AKAR	PKA activity	cpGFP	PKA substrate/FHA1 from AKAR	400/509	515	185% (in vivo)	[28]
ExRai-CKAR	PKC activity	cpGFP	PKC substrate/FHA1 from AKAR	400/509	515	155% (in vivo)	[28]
ExRai-AktAR	PKB activity	cpGFP	PKB substrate/FHA1 from AKAR	400/509	515	93% (in vivo)	[28]
yellow-sinphos	Insulin induced protein phosphorylation	cpCitrine	PH-PTB/SH2n	NM	NM	15% (in vivo)	[212]
sapphireAKAR	PKA activity	cp-T-sapphire	PKA substrate/FHA1 from AKAR	400	513	−25% (in vivo)	[28]
sapphireCKAR	PKC activity	cp-T-sapphire	PKC substrate/FHA1 from AKAR	400	513	−33% (in vivo)	[28]

The raw colors refer to the colors of fluorescent proteins used as reporter domains. NM—not measured. RA—response amplitude.

**Table 8:** GEFIs for neurotransmitters measuring.

Name	Analyte	Permutant	Sensor Unit	λex, nm	λem,nm	Kd	RA	Reference
SF-Azurite-iGluSnFR	Glutamate	cpAzurite	*E. coli* GltI	370	450	62 ± 11 μM (in vitro)	NM	[102]
iGluSnFR	Glutamate	cpGFP	*E. coli* GltI	NM	515	107 μM (in vitro)	450%	[84]
SF-iGluSnFR	Glutamate	cpSF-GFP	*E. coli* GltI	400	510	40 μM (in vitro)	NM	[102]
SF-iGluSnFR.A184S	Glutamate	cpSF-GFP	*E. coli* GltI	NM	NM	7 μM (in vitro)	NM	[102]
SF-iGluSnFR.S72A	Glutamate	cpSF-GFP	*E. coli* GltI	NM	NM	200 μM(in vitro)	NM	[102]
iGABASnFR	GABA	cpSF-GFP	Pf622	NM	NM	9 μM (in vitro)	250%	[85]
iGABASnFRF.102Y.Y137L	GABA	cpSF-GFP	Pf622	NM	NM	70 μM (in vitro)	350%	[85]
iGABASnFRF.102G	GABA	cpSF-GFP	Pf622	NM	NM	50 μM (in vitro)	450%	[85]
dLight1.1	Dopamine	cpGFP	DRD1	NM	516	330 nM	230%	[82]
dLight1.2	Dopamine	cpGFP	DRD1	NM	516	770 nM	340%	[82]
dLight1.3a	Dopamine	cpGFP	DRD1	NM	NM	2.3 μM	660%	[82]
dLight1.3b	Dopamine	cpGFP	DRD1	NM	NM	1.68 μM	930%	[82]
dLight1.4	Dopamine	cpGFP	DRD4	NM	NM	4.1 nM	170%	[82]
dLight1.5	Dopamine	cpGFP	DRD2	NM	NM	110 nM	180%	[82]
B1AR-cpGFP	β-1 adrenergic receptors agonists	cpGFP	B1AR	NM	NM	NM	70%	[82]
B2AR-cpGFP	β-2 adrenergic receptors agonists	cpGFP	B2AR	NM	NM	NM	150%	[82]
KOR-cpGFP	κ-type opioid receptors agonists	cpGFP	KOR	NM	NM	NM	80%	[82]
MOR-cpGFP	μ-type opioid receptors agonists	cpGFP	MOR	NM	NM	NM	60%	[82]
A2AR-cpGFP	α-2 adrenergic receptors agonists	cpGFP	A2AR	NM	NM	NM	40%	[82]
5HT2A-cpGFP	Serotonin receptor-2A agonists	cpGFP	5HT2A	NM	NM	NM	40%	[82]
MT2-cpGFP	Melatonin type-2 receptor agonists	cpGFP	MT2	NM	NM	NM	40%	[82]
GACh1.0	Acetylcholine	cpGFP	M3	NM	NM	NM	30%	[83]
GACh1.5	Acetylcholine	cpGFP	M3	NM	NM	NM	70%	[83]
GACh2.0	Acetylcholine	cpGFP	M3	NM	NM	EC50 = 0.7 μM	90%	[83]
GRAB_DA1m_	Dopamine	cpEGFP	DRD2	NM	NM	EC50 = 130 nM	90%	[88]
GRAB_DA1h_	Dopamine	cpEGFP	DRD2	NM	NM	EC50 = 10 nM	90%	[88]
GRAB_NE1m_	Norepinephrine	cpEGFP	A2AR	NM	NM	930 nM	230%	[89]
GRAB_NE1h_	Norepinephrine	cpEGFP	A2AR	NM	NM	83 nM	130%	[89]
SF-Venus-iGluSnFR	Glutamate	cpVenus	*E. coli* GltI	510	520	27 ± 2 μM (in vitro)		[102]
R-iGluSnFR1	Glutamate	cpmApple	*E. coli* GltI	562	588	11 μM (in vitro)	4.9-fold	[58]

The raw colors refer to the colors of fluorescent proteins used as reporter domains. Kd—dissociation constant. NM—not measured. RA—response amplitude.
